# Rewarming From Hypothermic Cardiac Arrest Applying Extracorporeal Life Support: A Systematic Review and Meta-Analysis

**DOI:** 10.3389/fmed.2021.641633

**Published:** 2021-05-13

**Authors:** Lars J. Bjertnæs, Kristian Hindberg, Torvind O. Næsheim, Evgeny V. Suborov, Eirik Reierth, Mikhail Y. Kirov, Konstantin M. Lebedinskii, Torkjel Tveita

**Affiliations:** ^1^Anesthesia and Critical Care Research Group, University of Tromsø (UiT), The Arctic University of Norway, Tromsø, Norway; ^2^K. G. Jebsen Thrombosis Research and Expertise Center, University of Tromsø (UiT), The Arctic University of Norway, Tromsø, Norway; ^3^Cardiovascular Research Group, Department of Clinical Medicine, Faculty of Health Sciences, University of Tromsø (UiT), The Arctic University of Norway, Tromsø, Norway; ^4^The Nikiforov Russian Federation Center of Emergency and Radiation Medicine, St. Petersburg, Russia; ^5^Science and Health Library, University of Tromsø, The Arctic University of Norway, Tromsø, Norway; ^6^Department of Anesthesiology and Intensive Care, Northern State Medical University, Arkhangelsk, Russia; ^7^Department of Anesthesiology and Intensive Care, North-Western State Medical University Named After I. I. Mechnikov, St. Petersburg, Russia; ^8^Federal Research and Clinical Center of Intensive Care Medicine and Rehabilitology, Moscow, Russia; ^9^Division of Surgical Medicine and Intensive Care, University Hospital of North Norway, Tromsø, Norway

**Keywords:** cardiac arrest, cardiopulmonary bypass, extracorporeal membrane oxygenation, hypothermia, hypothermic cardiac arrest, resuscitation, rewarming, extracorporeal life support

## Abstract

**Introduction:** This systematic review and meta-analysis aims at comparing outcomes of rewarming after accidental hypothermic cardiac arrest (HCA) with cardiopulmonary bypass (CPB) or/and extracorporeal membrane oxygenation (ECMO).

**Material and Methods:** Literature searches were limited to references with an abstract in English, French or German. Additionally, we searched reference lists of included papers. Primary outcome was survival to hospital discharge. We assessed neurological outcome, differences in relative risks (RR) of surviving, as related to the applied rewarming technique, sex, asphyxia, and witnessed or unwitnessed HCA. We calculated hypothermia outcome prediction probability score after extracorporeal life support (HOPE) in patients in whom we found individual data. *P* < 0.05 considered significant.

**Results:** Twenty-three case observation studies comprising 464 patients were included in a meta-analysis comparing outcomes of rewarming with CPB or/and ECMO. One-hundred-and-seventy-two patients (37%) survived to hospital discharge, 76 of 245 (31%) after CPB and 96 of 219 (44 %) after ECMO; 87 and 75%, respectively, had good neurological outcomes. Overall chance of surviving was 41% higher (*P* = 0.005) with ECMO as compared with CPB. A man and a woman had 46% (*P* = 0.043) and 31% (*P* = 0.115) higher chance, respectively, of surviving with ECMO as compared with CPB. Avalanche victims had the lowest chance of surviving, followed by drowning and people losing consciousness in cold environments. Assessed by logistic regression, asphyxia, unwitnessed HCA, male sex, high initial body temperature, low pH and high serum potassium (s-K^+^) levels were associated with reduced chance of surviving. In patients displaying individual data, overall mean predictive surviving probability (HOPE score; *n* = 134) was 33.9 ± 33.6% with no significant difference between ECMO and CPB-treated patients. We also surveyed 80 case reports with 96 victims of HCA, who underwent resuscitation with CPB or ECMO, without including them in the meta-analysis.

**Conclusions:** The chance of surviving was significantly higher after rewarming with ECMO, as compared to CPB, and in patients with witnessed compared to unwitnessed HCA. Avalanche victims had the lowest probability of surviving. Male sex, high initial body temperature, low pH, and high s-K^+^ were factors associated with low surviving chances.

## Introduction

Accidental hypothermia (AH) is an unintended drop in body core temperature to below 35°C, due to exposure to cold environments or a decrease in metabolic rate. The condition is characterized by different stages of severity, as mild AH from 35 to 32°C, moderate AH from 32 to 28°C, severe AH below 28°C and deep AH below 20°C, based on the prevailing core temperature (1).

In 1968, Fell et al. reported the successful rewarming and resuscitation from HCA of a pentobarbital-poisoned woman by means of cardiopulmonary bypass (CPB) between a femoral vein and artery. She was discharged from hospital with no sequela (2). In the ensuing years, rewarming by extracorporeal life support (ECLS) became “the gold standard” for rewarming of patients from HCA. However, it was not until 2011 that Morita et al. as the first, showed the sovereignty of ECLS to conventional invasive rewarming in patients with maintained circulation and in victims of HCA alike. Compared with other rewarming modalities, ECLS also ensures blood oxygenation and organ perfusion, in addition to core rewarming (3). The latter results were recently confirmed in a nationwide Japanese study by Ohbe et al. who reported a risk reduction of 8.3 % (CI 95% 1.9–15%) in an ECMO group compared with a conventional cardiopulmonary resuscitation (CPR) group (4).

Introduction of ECLS with biocompatible membranes causing less contact activation of the complement system and the coagulation and the fibrinolytic systems (5–7), led to increased use of extracorporeal membrane oxygenation (ECMO) for rewarming of HCA patients. With the emergence of the heparin coating of oxygenator membranes, it became possible both to resuscitate victims of HCA and, if necessary, to use ECMO for heart and lung support for weeks postoperatively. In a multivariate analyses comparing victims of HCA rewarmed with CPB or ECMO, Ruttmann et al. suggested that those rewarmed with ECMO had a more than six-fold higher probability of surviving compared with those treated with CPB (8).

Of note, the rate of survival from HCA differed significantly between patients who were able to breathe during cooling and those who additionally were exposed to asphyxia due to drowning or burial by snow or soil before cardiac arrest (9–11). Apparently, the prognosis of HCA also depended on the cardiorespiratory condition, the circumstances causing the cooling and the treatment given from the scene of accident to a center where resuscitation with ECLS could take place (12). However, if such a center should be out of reach, for instance, because of too poor weather conditions for helicopter evacuation, a rewarming rate of 6.8°C/h (13) and a successful outcome of HCA has been reported even after 6 h and 30 min of manually performed CPR (14).

In deep AH, nearly all patients are found with asystole (15–17) and those with body core temperatures below 32°C after severe trauma, have an almost 100% mortality rate (18). Dunne et al. reviewed the results of applying extracorporeal circulation for rewarming of patients with HCA, and reported a survival rate of nearly 6 % after pure HCA (19). By contrast, in those who suffered hypoxic/asphyctic HCA, survival rate reached only 23.4%, whereof 9.4% survived with good neurological outcome. The investigators unequivocally recommended ECLS for rewarming of patients with HCA, albeit with some precautions, particularly with regard to the serum s-K^+^ level, which traditionally be considered as the single most important factor for predicting survival from HCA (19). Recently an international group of investigators worked out a hypothermia outcome prediction after extracorporeal life support (HOPE) score, based on four continuous and two categorical variables identified in HCA patients upon admission to hospital (20). The latter reported an average survival rate of 37%, whereof 84% had a “favorable neurological outcome” corresponding to Cerebral Performance Category (CPC) of 1 or 2 at hospital discharge (21). As assessed by receiver operating characteristic (ROC), the HOPE score had an AUC of 0.895, which significantly outperformed the outcome predictive value of serum s-K^+^ alone (AUC of 0.774) (20). Moreover, Saczkowski et al. found a survival rate of good outcome of 40.3 %, but in their analyses of 658 victims of HCA or severe cardiovascular instability, data were from 44 observational studies and 40 case reports combined (22).

Aims of this review and meta-analyses were firstly, to assess survival to hospital discharge after rewarming from HCA employing ECLS; secondly, to find out whether the probability of surviving to hospital discharge differed depending on whether the patients underwent rewarming with CPB or/and ECMO. We also assessed whether the chance of surviving differed between sexes, and whether neurological outcome, classified as good or bad at hospital discharge depended on the ECLS rewarming technique that had been applied.

## Methods

**Figure 1:**
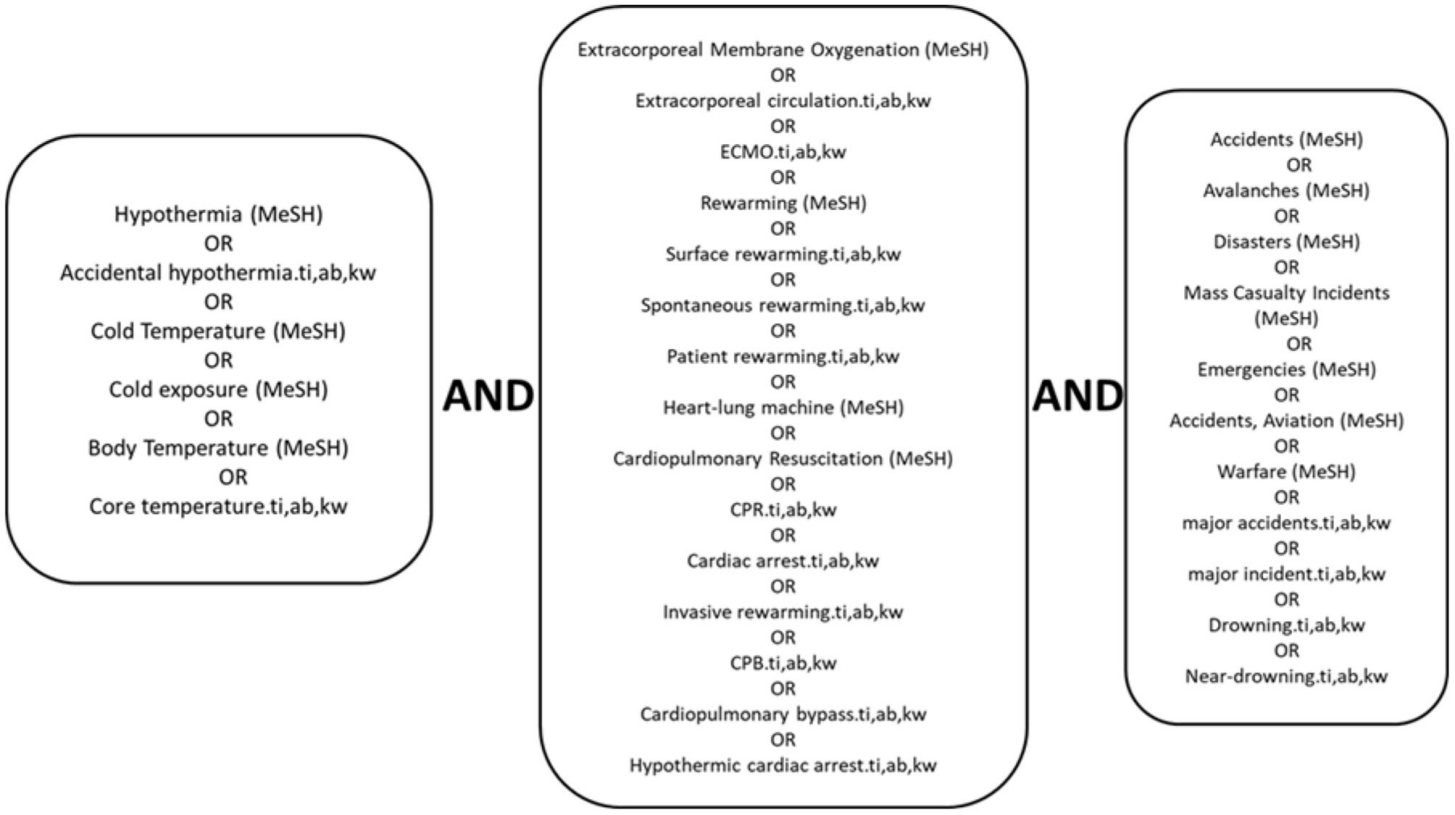
Search strategy. Systematic literature search of August 2020 in the following databases: Ovid MEDLINE^(R)^ In-Process & Other Non-Indexed Citations. Ovid MEDLINE^(R)^ Daily and Ovid MEDLINE^(R)^ 1946 to Present and Embase Classic+Embase 1947 to Present. Medline subject headings; ti, titles; ab, abstracts; kw, keywords.

We performed this systematic review and meta-analysis on the basis of a protocol published in PROSPERO international prospective register of systematic reviews, registration no. 47,934. We undertook a systematic literature search in April 2016, which was updated 28.08.2020. The search was limited to references with, at least, an abstract in English, French, or German (Figure 1). The search strategy was in accordance with the Preferred Reporting Items for Systematic Reviews and Meta-Analyses (PRISMA) in the following databases: Ovid MEDLINE^(R)^ and In-Process & Other Non-Indexed Citations and Daily, 1946 to present, and Embase Classic^+^Embase, 1947 to present. We used the controlled vocabulary of Medical Subject Headings (MeSH) from MEDLINE, and the Emtree thesaurus from EMBASE when applicable. In addition, we searched the search fields, title, abstract and keywords and scanned reference lists of included articles for references of interest. We exported all references to Endnote TM (97.4; Thompson Reuters. Toronto, ON, Canada) removing duplicates. Since the review is limited to a study of published literature, approval by the Regional Committee on Human Research Ethics was not required.

### Inclusion Criteria

- Retrospective observational studies of patients with HCA, who underwent attempted rewarming by means of CPB or/and ECMO, published in peer reviewed international journals. If necessary, we contacted the authors for additional information about survivors and non-survivors, for which we thanked them in the Acknowledgments.
- Review articles on rewarming from HCA and other articles elucidating this special field of research will be referred to in Introduction and Discussion without being included in the meta-analysis.
- Case reports of HCA of three patients or less will be presented in a special chapter and additionally as abbreviated medical records in Appendix without being included in the meta-analysis.

All reports accessible on Medline and EMBASE about victims of HCA after varying etiologies of exposure to cold, such as burial by avalanches, near-drowning or drowning, exposure to cold subsequent to major trauma, mountaineers falling into a crevasse, or urban cases of people found outdoors after intoxication, were eligible for the study. The patients had in common that they had undergone pre-hospital–and/or intrahospital CPR and attempted rewarming by means of ECLS, using either CPB or ECMO, both equipped with a heat exchange device. CPB is traditionally used in cardiac surgery, most frequently, connected between the right atrium and the aorta, and is fitted with an oxygenator, a blood reservoir and a suctioning system for drainage of the left ventricle. CPB can also be connected between peripheral vessels, most frequently between a femoral vein–and artery. The ECMO system consists only of a membrane oxygenator and a roller pump or centrifugal pump connected in a closed loop between a vein and artery, most frequently, a femoral vein and artery. In earlier days, CPB was used routinely, and most frequently with access via the femoral vein and artery (VA) thereby enabling simultaneous CPR until extracorporeal circulation was established. Occasionally, and most often in small children, CPB or ECMO is established with access via a sternum split. Recently, investigators introduced a miniaturized portable percutaneous cardiopulmonary bypass (PPCPB) version of the ECMO-system (23).

In some patients, the investigators started rewarming with CPB, but experienced that the patient was difficult to wean off because of cardiopulmonary failure and subsequently connected him or her to VA ECMO for heart and/or lung support for hours or days until obtaining sufficient recovery for weaning. We listed these patients as belonging to the ECMO group.

### Exclusion Criteria

- Reports of victims of AH with maintained circulation on admittance to hospital not requiring rewarming with ECLS.
- Reports of patients with HCA secondary to terminal stage of malignant disease, or with s-K^+^ > 12 mmol/L.

#### Primary Study Objectives

- Number and percentage of patients with HCA discharged from hospital alive after attempted rewarming with CPB or/and ECMO.

#### Other Study Objectives

- Number and percentage of patients surviving with good or poor neurological outcome depending on the applied rewarming technique. When available, we listed Glasgow-Pittsburgh Cerebral Performance Categories scale (CPC) 1 and 2 as good neurological outcomes and CPC 3 and 4 as poor neurological outcome (21).

Comparing the probability of surviving:

- between male and female.
- between asphyctic and non-asphyctic victims of HCA.
- between victims of witnessed (including rescue collapse) and unwitnessed HCA.

### Data Analysis

#### Main Cohort

Bibliographic and demographic variables are presented as well as survival at discharge from hospital. Clinical and laboratory data were listed in tables as mean and standard deviation (SD) or median and range or interquartile range (IQR), as outlined in the individual studies. Odds ratio (OR) and relative risk ratio (RR) were calculated to assess the difference in survival and neurological outcome following HCA between patients that had been attempted rewarmed by means of CPB and/or ECMO.

#### Subset of Studies Displaying Individual Data

In this subset of studies, we compared initial body temperature, pH, s-K^+^, s-lactate, PaCO_2_, PaO_2_, and HOPE survival prediction probability score in percent between survivors and non-survivors, independent of resuscitation technique, and separately between patients treated with ECMO and CPB (20). HOPE score was calculated as 2.44 − 1.55*male* − 1.95 (*asphyxia related mechanism*) − 0.0191*age* − 2.07 (*potassium*) − 0.573 (*CPR duration*) + 0.937*temperature* − 0.0247*temperature*^2^. In this score, K^+^ was presented in mmol/L, CPR duration in minutes (min) and temperature in degrees Celcius (°C). We used the score in the subsequent formula to calculate HOPE predictive survival probability as a percentage = exp (score)/[1+exp (score)] × 100.

In this subset of the study, data were analyzed with SigmaPlot (version 14.0) graphing and statistical analysis software and presented as mean ± SD, or as median and IQR, depending on whether data were normally distributed or not, as assessed with the Shapiro-Wilks test. We performed receiver operator characteristic (ROC) curve analysis of HOPE score and serum K^+^ concentration separately, using the MedCalc software to compare them as predictors of survival probability (24, 25). We used Welch's *t*-test or Mann–Whitney *U*, as appropriate for comparison between groups with unequal variances. We also calculated OR per SD or unit change in clinical and laboratory variables by means of logistic regression using the R Project for Statistical Computing (version 4.0.1).

#### Case Reports

We present abbreviated medical records from case reports in Appendix without including them in the meta-analysis.

## Results

### Literature Search

**Figure 2:**
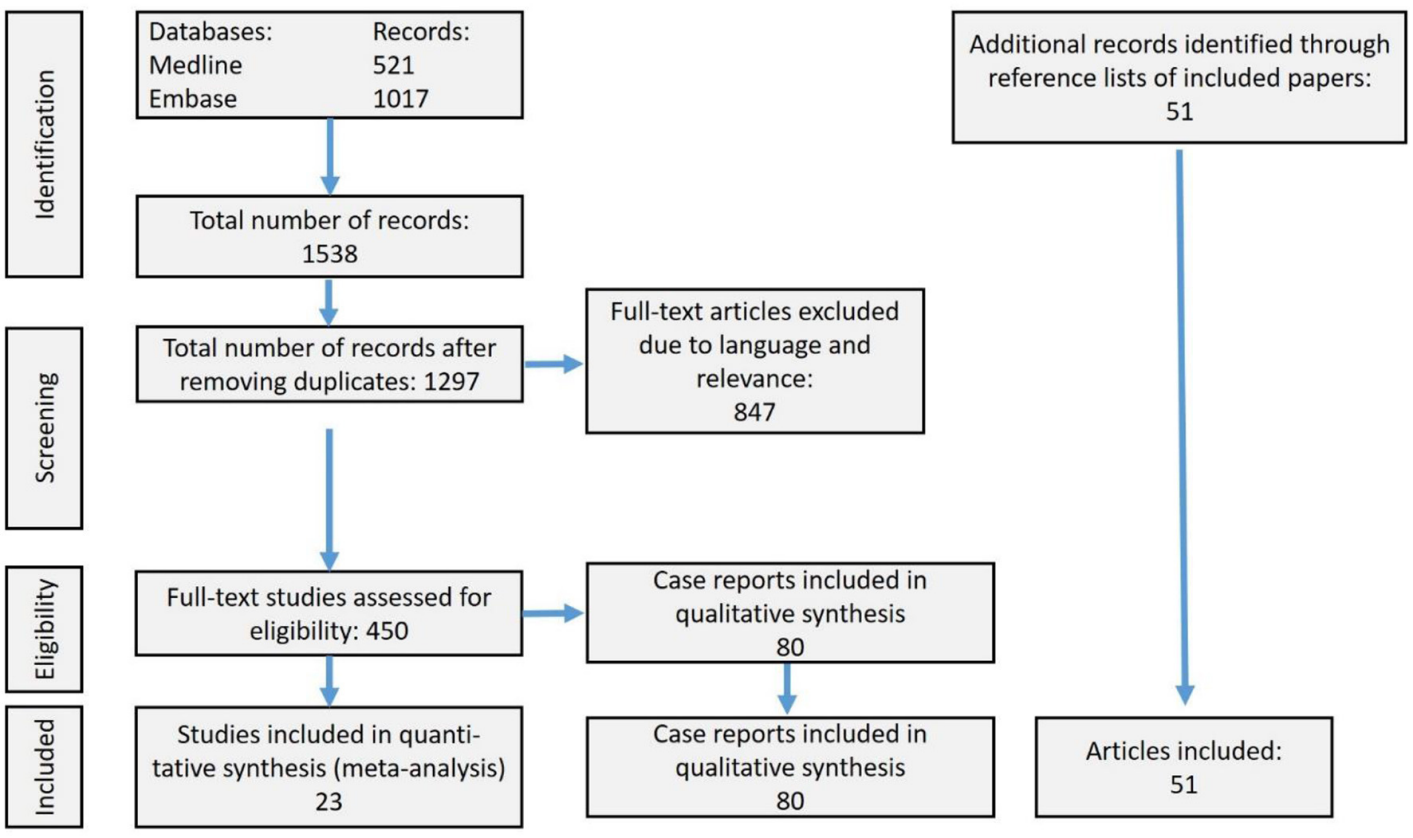
Prisma flow-chart displaying the literature search history. Of totally 1,538 references, we included 23 observational studies of patients with hypothermic cardiac arrest, who underwent attempted resuscitation with extracorporeal life support (ECLS) in the meta-analysis. Fifty-one articles lead up to the present review and meta-analysis and/or were used for discussion of our findings. Finally, we addressed 80 case reports.

From a total of 1,538 articles considered, 1,297 were included in the present review and meta-analysis after removing duplicates (Figure 2). After excluding 847 full text articles due to relevance or language, 450 remaining references were assessed for eligibility. We found 23 observational studies eligible for the meta-analysis and 51 articles describing the development of the field up to present. The latter articles also were referred to when discussing our findings. We also assessed 80 case reports with medical records of 96 victims rewarmed from HCA, but without including them in the meta-analysis.

### Main Cohort

Each of the 23 included studies consisted of from four to 68 victims of HCA, who underwent attempted rewarming with ECLS. Table 1 displays year of publication, demographic data and lowest body temperatures in survivors and non-survivors.

**Table 1:** Bibliographic and demographic data and lowest core temperatures reported in case observation studies of totally 464 victims of hypothermic cardiac arrest (HCA), who underwent attempted rewarming with ECLS (CPB or ECMO).

**References**	**Period**	**HCA**	**Sex**		**Age**		**Lowest core temperature**	
	**Years**	**Victims**	**Male**	**Female**	**Survivors**	**Non-survivors**	**Survivors**	**Non-survivors**
	**(from–to)**	**(*N*)**	**(*n*)**	**(*n*)**	**(years)**	**(years)**	**(°C)**	**(°C)**
Splittgerber et al. (26)	1980–1986	6	6	0	54 (r40–55)	40 (r34–40)	22 (r20.9–24.4)	22.8 (r21.6–24.4)
Brunette and McVaney (27)	1988–1999	10	8	2	36.5 (r38–48)	58 (r11–88)	26.2 (r20.6–30.3)	26.3 (r26.1–26.5)
Mair et al. (28)	<1993	22	19	3	14 (r5–23)	28 (r3–54)	20.3 (r16.5–24)	24.5 (r22–30)
Letsou et al. (29)	1992	5	4	1	12 (r6–57)	56 (r40–52)	24.4 (r22–25)	24.3 (r22.5–26)
Locher et al. (10)^*^	1980–1987	17	13	4				
Walpoth et al. (30)	1977–1993	15	8	7	25.2 ± 9.9		21.8 ± 2.5	
Hauty et al. (31)	May 1986	11	5	6	15 (r15–15)	15 (r15–41)	22.7 (r22–23.4)	7 (r1–19.7)
Ruttmann et al. (8)	1987–2006	59	49	10	38.3 ± 20.1	29.5 ± 16.7	24.2 ± 0.35	24.5 ± 0.68
Wanscher et al. (32)	11.02.2011	7	5	2	16 (r15–45)		18.4 (r15.5–20.2)	
Silfvast and Pettila (33)	1991–2000	23	19	4	41.5 (IQR32.5–53.2)	58 (IQR55.5–68)	23.5 (r22.4–25.6)	26 (r22–29.8)
Morita et al. (3)	1992–2009	6						
Schober et al. (34)	1991–2010	9	5	4	50 (r18–67)	52 (r31–63)	24 (r22.7–25.3)	22.9 (r22.6–25.1)
Weuster et al. (35)	2003–2012	8	6	2	75	7 (r3.5–58)	21.6	19 (r13.4–29.4)
Moroder et al. (36)	2008–2013	4	4	0		44.5 (r39–56)		26.8 (r22–28)
Boue et al. (37)	1994–2013	19	16	3	36 (r17–41)	32.5 (r15–62)		
Sawamoto et al. (38)	1994–2012	26	18	8			24.4 ± 3.47	
Debaty et al. (39)	2003–2012	23	15	8	46 (r17–75)	32 (r2–70)	24 (r16.3–28)	25.3 (r18.3–28)
Champigneulle et al. (40)	2002–2012	20	15	5	44 ± 14	42 ± 12	21 ± 4	27 ± 3
Hilmo et al. (12)	1984–2013	35	24	11			26.8 (r13.7–32.9)	23.4 (r8.9–30)
Darocha et al. (41)	2013–2015	10	7	3	52 (r25–78)	45 (r28–54)	24 (r16.9–25.4)	25.9 (r22–29)
Svendsen et al. (42)	1987–2015	68	52	16	32 (r1.5–76)	30 (r1.7–76)	22 (r19–27)	25 (r17–34)
Khorsandi et al. (43)	2009–2016	10	6	4	51 (r26–65)	70 (r55–73)	19.8 (r18–22)	23 (r22–24)
Ruttmann et al. (44)	2004–2016	51	37	14				

*IQR, interquartile range; r, range; N, total number of patients studied; n, number of patients in subgroups;*

* *Number of men and women estimated by Locher et al. (10)*.

Of totally 464 victims of HCA, 345 were men and 119 were women, as shown in Table 2. Two-hundred and nineteen patients underwent attempted rewarming with ECMO and 245 with CPB. Independent of rewarming technique, 291 of the patients (63%) had transient return of spontaneous circulation (ROSC). Of the whole cohort, 172 patients (37%) were discharged from hospital alive, 96 patients (44%) survived after rewarming with ECMO and 76 patients (31%) after CPB. Overall, 80% of the survivors had good neurological outcomes, 75% after ECMO-treatment and 87% after CPB (not displayed in tables).

**Table 2:** Survival of totally 464 victims of hypothermic cardiac arrest sorted by gender and ECLS rewarming technique (CPB or ECMO) in 23 observational studies.

		**CPB**						**ECMO**					
		**Both sex (*****n*****)**		**Male (*****n*****)**		**Female (*****n*****)**		**Both sex (*****n*****)**		**Male (*****n*****)**		**Female (*****n*****)**	
**References**	**Patients (*N*)**	**Surv**.	**Pat**.	**Surv**.	**Pat**.	**Surv**.	**Pat**.	**Surv**.	**Pat**.	**Surv**.	**Pat**.	**Surv**.	**Pat**.
Splittgerber et al. (26)	6	3	6	3	6	0	0						
Brunette and McVaney (27)	10	6	10	4	8	2	2						
Mair et al. (28)	22	2	22	1	19	1	3						
Letsou et al. (29)	5	3	5	2	4	1	1						
Locher et al. (10); Walpoth et al. (30)	32	15	32	8	21	7	11						
Hauty et al. (31)	11	2	11	1	5	1	6						
Ruttmann et al. (8)	34	3	34	2	28	1	6						
Ruttmann et al. (8)	25							9	25	6	21	3	4
Wanscher et al. (32)	7							7	7	5	5	2	2
Silfvast and Pettilla (33)	23	14	23	11	19	3	4						
Schober et al. (34)	9							5	9	2	5	3	4
Darocha et al. (41)	10							7	10	5	7	2	3
Morita et al. (3)	6							5	6	4	4	1	2
Weuster et al. (35)	8							1	8	1	6	0	2
Moroder et al. (36)	4							0	4	0	4	0	0
Boue et al. (37)	19							3	19	2	16	1	3
Debaty et al. (39)	23							9	23	3	15	6	8
Sawamoto et al. (38)	26							26	26	18	18	8	8
Champigneulle et al. (40)	20							2	20	0	15	2	5
Hilmo et al. (12)	29	6	29	4	20	2	9						
Hilmo et al. (12)	6							4	6	2	4	2	2
Svendsen et al. (42)	65	17	65	9	49	8	16						
Svendsen et al. (42)	3							1	3	1	3		
Khorsandi et al. (43)	8	5	8	2	5	3	3						
Khorsandi et al. (43)	2							2	2	1	1	1	1
Ruttmann et al. (44)	51							15	51	10	37	5	14
Totally	464	76	245	47	184	29	61	96	219	60	161	36	58

*Surv, Survivor; pat, patient; N, total number of patients in each study; n, number of patients in subgroups*.

### Probability of Surviving HCA Depending on Sex and ECLS Technique

By including all the survivors and non-survivors of HCA from Table 2, we estimated RR of surviving with ECMO vs. CPB as 1.41 (CI 95% 1.11–1.80; *P* = 0.005). The chance of surviving with ECMO vs. CPB for each sex separately, displayed an estimated RR of 1.46 (CI 95% 1.06–2.00; *P* = 0.043) for a man and 1.31 (CI 95% 0.94–1.82; *P* = 0.115) for a woman. Moreover, the likelihood of surviving HCA for a woman as compared to a man, both techniques considered together, revealed a RR of 1.76 (CI 95% 1.40–2.21; *P* = 0.0007). Considering the rewarming techniques separately, the chance of surviving HCA for a woman as compared to a man was estimated to a RR of 1.67 (CI 95% 1.25–2.21; *P* = 0.0004) after ECMO and of 1.86 (CI 95% 1.30–2.67; *P* = 0.0007) after CPB.

Calculation of the chance of surviving with good vs. poor neurological outcome with ECMO vs. CPB, resulted in RR of 0.86 (CI 95% 0.75–0.99; *P* = 0.047), indicating 14 % less probability of a good outcome after ECMO. We also estimated RR of surviving with poor outcome vs. dying to 2.92 (CI 95% 1.44–5.91; *P* = 0.003) after treatment with ECMO in comparison with CPB.

Additional data presented in Table A1 shows incidents that caused HCA, initial ECG rhythms, duration of CPR until commencement of rewarming with CPB or/and ECMO, in survivors and non-survivors, respectively, and number of patients, who developed lung edema. Moreover, Table A2 depicts s-K^+^, s-pH and s-lactate, distinguishing between survivors and non-survivors.

### Subset of Studies Displaying Individual Data

Fifteen studies consisting of totally 200 victims of HCA presented individual data, of whom 77 patients (38.5%) survived to hospital discharge after successful rewarming. Forty-one of 93 patients (44 %) survived after rewarming with ECMO and 36 of 107 patients (34%) after CPB.

Relative risk estimates (Table 3) with survival as outcome and avalanche as reference category, showed that avalanche victims had the lowest chance of surviving. Survival probability was greater after submersion, and following exposure to cold with a possibility of breathing during gradual cooling prior to HCA. Table A3 surveys initial core temperature, pH, s-K^+^, s-lactate, PaCO_2_, and PaO_2_ in survivors and non-survivors, respectively, in the subset of studies displaying individual data. Concerning these variables, we found statistically significant differences between survivors and non-survivors, but no significant differences depending on the ECLS technique used for rewarming.

**Table 3:** Risk ratios of victims of hypothermic cardiac arrest with survival as outcome and avalanche as reference category.

**Cause of HCA**	**Survivors**	**Non-survivors**	**Risk ratio**	***P*–value**
Avalanche	9	44	1 (ref)	
Submersion. Child	5	21	1.13	1.00
Submersion adult	15	24	2.26	0.030
Exposure	47	35	3.38	2.72e-06

*P-values are from Fisher exact tests. HCA, hypothermic cardiac arrest*.

### Non-asphyctic vs. Asphyctic HCA

The calculated probability of surviving non-asphyctic HCA (water immersion, trapped in crevasse, falling asleep outdoors)–vs. asphyctic HCA (buried by avalanche, drowning) for the whole cohort, resulted in RR of 2.45 (CI 95% 1.67–3.59; *P* = 0.0001). Correspondingly, RR were 2.25 (CI 95% 1.35–3.73; *P* = 0.004) and 2.54 (CI 95% 1.47–4.40; *P* = 0.0008) for men and women, respectively.

### Witnessed vs. Non-witnessed HCA

One hundred and forty eight patients had non-witnessed HCA and 50 of them survived. Correspondingly, 52 patients had a history of witnessed HCA or rescue collapse, whereof 26 were discharged from hospital alive, resulting in estimated RR of 1.48 (CI 95% 1.04–2.11; *P* = 0.03). Considering men and women separately, estimated RR of surviving witnessed vs. non-witnessed HCA were 1.47 (CI 95% 0.89–2.44; *P* = 0.1341) and 1.38 (CI 95% 0.91–2.09; *P* = 0.1271), respectively.

### Clinical and Laboratory Variables Assessed by Logistic Regression

**Figure 3:**
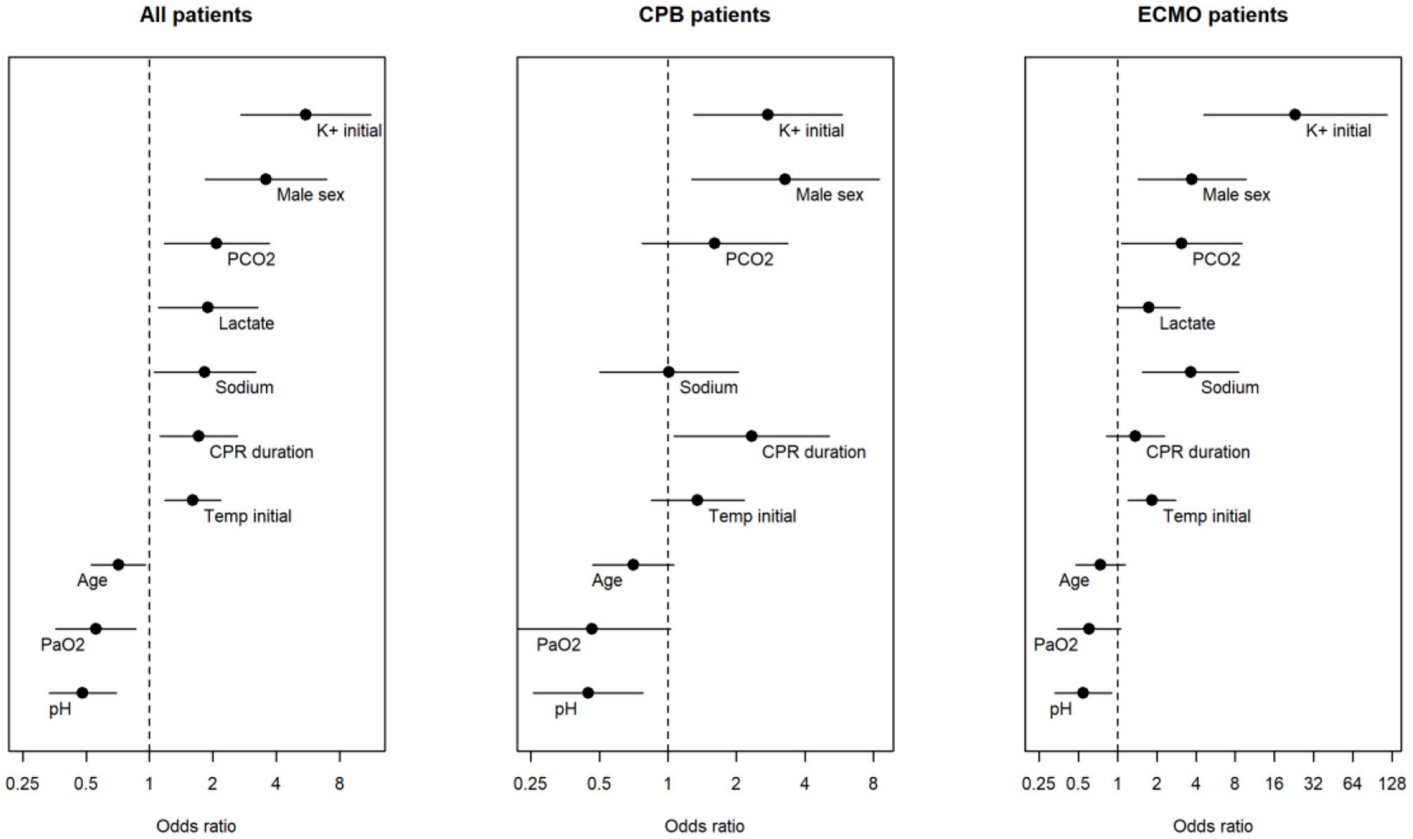
Odds ratios of different variables from univariate logistic regression with death as outcome. From left to right, results from all patients from whom we had access to individual data, who underwent attempts on resuscitation by means of cardiopulmonary bypass (CPB) or extracorporeal membrane oxygenation (ECMO), respectively. Male sex and initial body temperature are per unit increase, while the rest are per standard deviation increase.

Figure 3 depicts logistic regression of odds ratio per standard deviation (SD) or unit increase in clinical and laboratory variables in all victims of HCA at risk of dying following attempts on rewarming by means of CPB or ECMO.

Table 4 shows highly significant (*P* ≤ 0.001) positive correlations between s-lactate and initial s-K^+^. Positive correlations (*P* < 0.01) were also shown between concentrations of s-sodium (s-Na^+^) and s-lactate and between male sex and s-Na^+^ and initial s-K^+^ concentrations, and between the latter and the duration of CPR (*P* < 0.05). Moreover, significant negative correlations existed between pH and s-lactate (*P* < 0.001), between PaCO_2_ and PaO_2_ (*P* < 0.01) and between PaO_2_ and s-K^+^.

**Table 4:** Correlations between clinical and laboratory variables of a subset of victims of hypothermic cardiac arrest displaying individual data undergoing attempted rewarming by means of ECSL (ECMO or/and CPB).

	**Age**	**Male sex**	**Temp. initial**	**Sodium**	**Lactate**	**pH**	**PaCO_**2**_**	**PaO_**2**_**	**K^+^ initial**	**CPR duration**
Age	1	0.09	0.13	−0.01	−0.26^*^	0.32^***^	−0.17	−0.02	−0.18^*^	0.02
Male sex	0.09	1	0.11	0.24^*^	0.16	−0.16^*^	0.15	−0.12	0.22^*^	0.09
Temp. initial	0.13	0.11	1	0.07	0.10	−0.05	0.09	−0.01	0.02	−0.10
Sodium	−0.01	0.24^*^	0.07	1	0.48^**^	−0.21	0.26^*^	−0.23^*^	0.19	0.26^*^
Lactate	−0.26^*^	0.16	0.10	0.48^**^	1	−0.61^***^	0.42^**^	−0.19	0.47^***^	0.07
pH	0.32^***^	−0.16^*^	−0.05	−0.21	−0.61^***^	1	−0.43^***^	0.21^*^	−0.46^***^	−0.03
PaCO_2_	−0.17	0.15	0.09	0.26^*^	0.42^**^	−0.43^***^	1	−0.44^***^	0.46^***^	0.12
PaO_2_	−0.02	−0.12	−0.01	−0.23^*^	−0.19	0.21^*^	−0.44^**^	1	−0.23^*^	−0.24^*^
K^+^ initial	−0.18^*^	0.22^**^	0.02	0.19	0.47^**^	−0.46^***^	0.46^***^	−0.23^*^	1	0.21^*^
CPR duration	0.02	0.09	−0.10	0.26^*^	0.07	−0.03	0.12	−0.24^*^	0.21^*^	1

*Temp. Initial temperature; CPR duration, duration of cardiopulmonary resuscitation*.

* *The correlation is significant at the P ≤ 0.05 level (two-tailed)*.

** *The correlation is significant at the P ≤ 0.01 level (two-tailed)*.

*** *The correlation is significant at the P ≤ 0.001 level (two-tailed)*.

Type of variable, number of individuals registered (n), SD, OR, and *P*-values of patients with available data, distinguishing between patients treated with CPB and ECMO, respectively, are shown in Table A4. In the whole subset of patients with individual data, OR of s-K^+^ of 5.53 (*P* < 0.000002) and of male sex of 3.58 (*P* < 0.0002), respectively, represented the greatest risk of not surviving attempted rewarming from HCA. Whether CPB or ECMO were used, OR for male sex was estimated to 3.28 (*P* = 0.013) and 3.74 (*P* = 0.006), respectively. In the whole cohort, OR of pH was 0.48 (*P* = 0.00009). Highest calculated OR for s-K^+^ of 23.2 (*P* = 0.00012) was found in the ECMO group.

### HOPE Score

In 134 of the patients displaying individual data, we calculated percent (%) predictive probability of surviving according to HOPE survival probability score. For the whole, mean HOPE score was 33.9 ± 33% with medians of 58.5% (48%) and 6.0% (27.3%) in survivors and non-survivors (*P* < 0.001), respectively.

**Figure 4:**
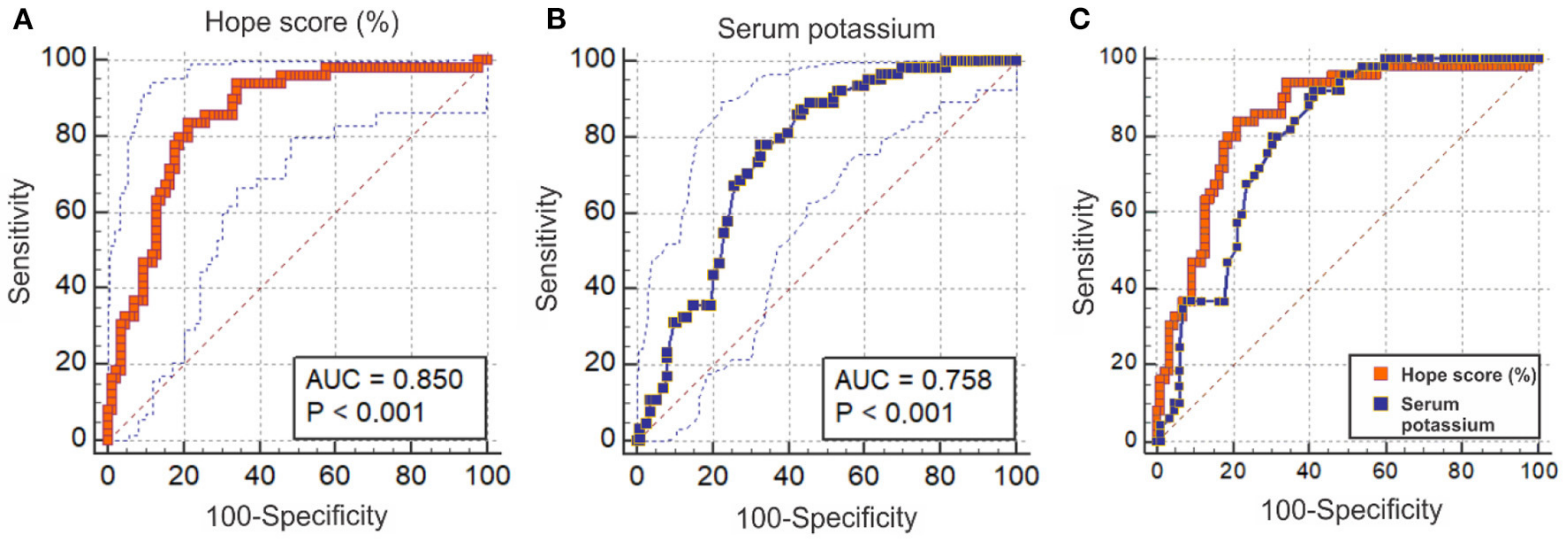
Predictive ability of surviving attempted resuscitation from hypothermic cardiac arrest assessed by ROC curves and AUC by HOPE score and serum K^+^. **(A)** displays Hope score (red) %. AUC 0.85 (CI 95% 0.78–0.91). **(B)** shows serum concentration of K^+^ (blue) predicting probability of surviving. AUC 0.79 (CI 95% 0.72–0.86). **(C)** depicts the difference between the AUC areas in curves **(A)** (red) and **(B)** (blue) of 0.056 (*P* = 0.0426).

We performed ROC curve analysis to compare HOPE score (*n* = 134) with s-K^+^ (*n* = 177) for predicting the probability of surviving HCA. Figures 4A,B present ROC curves and AUC for HOPE score and s-K^+^, respectively. Figure 4A shows Hope score AUC of 0.85 (CI 95% 0.78–0.91; *P* = 0.0001). Figure 4B shows ROC curve analysis of the predictive ability of surviving as assessed by s-K^+^, which resulted in an AUC of 0.76 (CI 95% 0.69–0.82). Figure 4C depicts ROC curves with a difference of 0.056 (*P* = 0.04) between AUC of Figures 4A,B.

### Case Reports

**Figure 5:**
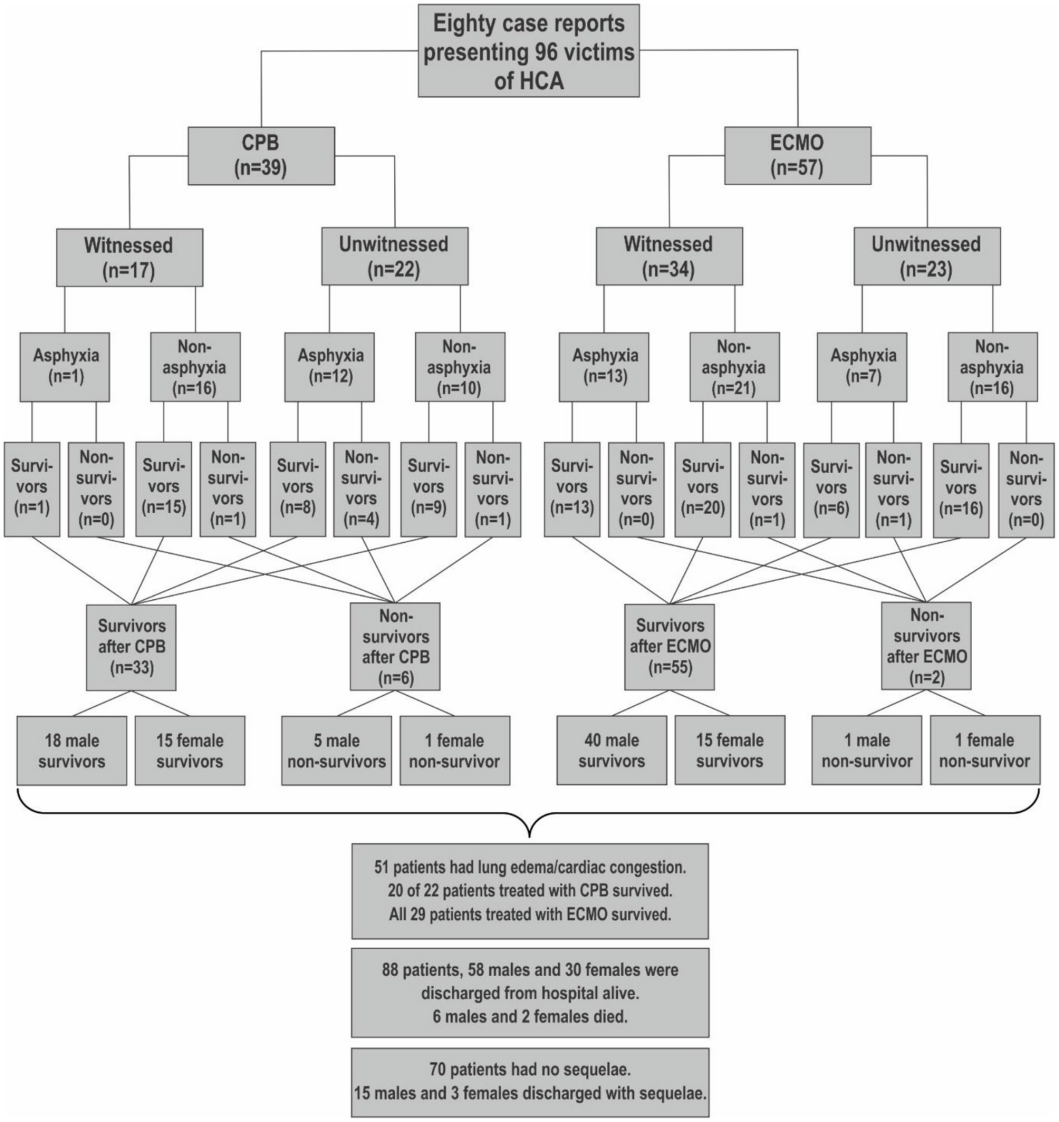
Flowchart surveying 80 case reports with 96 victims of hypothermic cardiac arrest (HCA) rewarmed with ECLS. Thirty-nine patients underwent resuscitation with cardiopulmonary bypass (CPB) and 57 patients with extracorporeal membrane oxygenation (ECMO). We distinguished between witnessed and not witnessed hypothermic cardiac arrest (HCA), whether hypothermia was associated with asphyctic incidents, as drowning or avalanche, or non-asphyctic incidents, as immersion in cold water or exposure to cold environments.

Figure 5 summarizes 80 case reports with 96 victims of HCA, who underwent rewarming with ECLS. Thirty-nine patients were rewarmed with CPB (2, 15, 45–75) and 57 patients with ECMO (15, 76–123). Overall 88 patients survived (92%). We distinguished between whether incidents causing HCA were witnessed (including rescue collapse) or not witnessed, whether HCA was preceded by asphyxia, or non-asphyxia with enabled breathing until HCA. The medical records are presented abbreviated in Table A5. Three men and one woman survived burial by avalanches for 6–7 h with no sequelae after rewarming with ECMO. Air pockets indicated that they had been breathing before HCA (66, 94, 95, 100). Fifteen men and eight women suffered HCA secondary to drug or alcohol abuse (2, 45, 46, 53, 54, 56, 57, 62, 63, 70, 76, 81, 86, 90, 91, 98, 101, 104, 105, 109, 121). All underwent successful resuscitation with ECLS. In 45 patients with an overall survival rate of 89%, and with the necessary data, we estimated mean predicted survival probability of 47 ± 28% by HOPE score.

Some reports focused on exceptionally low body temperatures (59, 82, 99, 123) prolonged CPR (70, 71, 91, 95, 100) and/or long distance to a hospital providing rewarming with ECLS. Recently, *a 2* ¼ *years old boy left home between 3 and 4 o'clock in the morning at an outdoor temperature of* −*5*°*C*. He *was found with HCA 620 m further down the street at 9 AM. Following CPR for 103 min, he was successfully rewarmed with ECMO, during which his body temperature fell from initially 12.6 to 11.8*°*C, the lowest ever recorded in a survivor of HCA. His predicted survival probability by HOPE score was 54%* (123). A reviewer made us aware of this case report from November 2020. Our last literature update was of ultimo August 2020.

## Discussion

### Main Cohort

The present systematic review and meta-analysis showed an overall survival rate from HCA of 37% after rewarming employing ECLS, which is consistent with that recently reported by Pasquier et al. (20). Of 172 survivors, 96 (56%) were discharged from hospital after ECMO treatment and 76 (44%) after CPB (Table 2). Relative risk assessment revealed that the probability of successful resuscitation was more than 40% greater after treatment with ECMO as compared with CPB. Of those undergoing successful rewarming, 75 and 87% survived with good neurological outcomes after reanimation with ECMO and CPB, respectively. The chance of surviving with a good rather than a poor neurological outcome was 14% less after ECMO as compared with CPB. On the other hand, the possibility of surviving with a poor neurological outcome rather than dying was almost 200% greater after ECMO as compared to CPB. This might be due to the fact that ECMO might bridge a period of poor cardiopulmonary function. In contrast, CPB is usually disconnected as soon as the patient has regained normal body temperature.

The assumed sovereignty of ECMO over CPB concerning survival from HCA, was primarily suggested by Ruttmann et al. These investigators noticed approximately a six-fold higher chance of surviving HCA after rewarming with ECMO as compared with CPB (8). Their findings were confirmed by Hilmo et al. (12). As deduced from their study, it was necessary to treat two HCA patients with ECMO to avoid one harmed patient after CPB (12, 124). Pasquier et al. in their large retrospective study also suspected a higher probability of surviving HCA after rewarming with ECMO in comparison with CPB, albeit the difference did not reach statistical significance (20). Although it was necessary to treat nearly 8 patients with ECMO to avoid one harmed patient after rewarming with CPB, our meta-analysis of 464 patients supported the superiority of ECMO as the method of choice for rewarming of victims of HCA.

Several reasons may account for the difference in outcome depending on whether patients with HCA underwent rewarming with ECMO or CPB. Technically, ECMO consists of a closed loop system with active venous drainage, which limits the size of the priming volume. Experiments on rats and piglets indicate that less hemodilution is associated with reduced activation of polymorph nuclear neutrophil granulocytes (125, 126). Smaller air-blood interface causes less contact activation in comparison with a traditional CPB (127). Furthermore, a heparin-coated membrane surface and no suctioning of spilt blood might reduce the inflammatory response to ECLS (127, 128). After rewarming, ECMO treatment has the advantage over CPB that it can continue for weeks, as a temporary support during cardiopulmonary failure (7, 129). In the main cohort consisting of 464 patients, we found 43 patients who were registered with cardiopulmonary failure or lung edema Table A1, but we do not know how many of these patients survived to hospital discharge, and if they survived because of ECMO treatment.

### Impact of Sex and ECLS Technique

Overall, women had more than 75% greater probability of surviving HCA, as compared to men. By considering each technique separately, women had a 67% higher chance of surviving after treatment with ECMO and 85% greater chance of surviving after CPB, as compared to men. The latter observation is concordant with Farstad et al. who argued that, women have more than an 80% higher chance of surviving HCA after rewarming with CPB as compared to men (9).

All ages taken into account, men have a six-fold higher probability of not surviving out of hospital cardiac arrest (OHCA), as compared to women. Particularly, premenopausal women had significantly higher rate of surviving OHCA as compared to men of the same age (130, 131). According to the latter investigators, the rate of favorable neurologic outcome was significantly higher in women aged 30 to 49 years than in men within the same age range. Apparently, female hormones, particularly estrogen, protects young women against fatal outcomes of OHCA (132). Wigginton et al. speculate that mitochondria-stabilizing effects of estrogen combined with anti-inflammatory and anti-apoptotic actions, turning off the cell death cascade, could explain these differences. The effects of drugs also might be different due to differences in bioavailability, distribution, metabolism, and elimination. Moreover, women have more compliant chest wall, possibly making CPR more effective (131). Nevertheless, in a potentially reversible condition like HCA, sex-dependent physiological and pharmacological differences need further elucidation.

### Subset of Studies Displaying Individual Data

#### Non-asphyctic vs. Asphyctic HCA

The chance of surviving depended on whether the patients became hypothermic before the outset of asphyxia and HCA. Locher et al. showed that the odds of surviving fell by 30 times in asphyctic patients (10) and other investigators recommend that victims with an indisputable history of asphyxia prior to cooling should not be rewarmed by CPB (9). In the subset of studies displaying individual data, the overall the chance of surviving was 145% higher in non-asphyctic patients as compared to asphyctic patients (*P* = 0.0001). As compared to their asphyctic counterparts, the probability of surviving was 154% higher in non-asphyctic women (*P* = 0.0008) and 125% higher (*P* = 0.004) in non-asphyctic men.

#### Witnessed—vs. Non-witnessed HCA

More than half a century ago, Negovskii demonstrated on hypothermic monkeys that particularly brisk handling could induce arrhythmias, like ventricular fibrillation or asystole (133). We found that the chance of being discharged from hospital alive increased by almost 50% after witnessed as compared with unwitnessed HCA (RR 1.48; *P* = 0.03), but no significant differences were found when focusing on men (*P* = 0.13) and women (*P* = 0.13) separately. According to a recent review by Frei et al. patients suffering from HCA due to rescue collapse had a survival rate of 73%, and nearly 90% of the survivors had a favorable neurological outcome (134). Moreover, Podsiadło et al. in a cohort of 221 patients with witnessed HCA observed an overall survival rate of 27%, whereof 83% had no neurologic deficit (135). Of note, Pasquier et al. in a univariate analysis of 237 victims of CA also found that witnessed CA was associated with better survival (*P* < 0.001). However, their multivariate analysis, with the necessary power, revealed that witnessed CA or not was not an independent predictor of survival in these patients (20). To avoid undetected rescue collapse, recent investigators recommend checking avalanche victims for vital signs, and monitor ECG from the outset of extrication (136). A higher rate of successful resuscitations after witnessed cardiac arrests agree with the findings of most investigators (8, 9, 30, 39, 44). Nevertheless, there are also reports of high survival rates in articles, in which information concerning whether CA is witnessed or not is lacking (33, 42).

#### Clinical–and Laboratory Variables Assessed by Logistic Regression

Logistic regression revealed that increasing s-K^+^, male sex, rising PaCO_2_, decreasing age, PaO_2_ and pH significantly increases the odds of dying from HCA, as displayed in Figure 3. High initial s-K^+^ -values and male sex were associated with the greatest risks of not surviving rewarming from HCA. Whether CPB or ECMO were used, s-K^+^, male sex, PaCO_2_ and duration of CPR were associated with the significantly highest OR for not surviving attempted rewarming from HCA. These data are specified in more detail in Table A3.

As shown in Table 4, we found highly significant (*P* ≤ 0.001) positive correlations between s-lactate and initial s-K^+^ concentrations. Positive correlations (*P* < 0.01) also were found between s-lactate and s-sodium concentrations, and between male sex, s-sodium and initial s-K^+^ concentrations (*P* < 0.05). Significant negative correlations existed between pH and s-lactate (*P* < 0.001), between PaCO_2_ and PaO_2_ (*P* < 0.01), and between PaO2 and s-K^+^ and the duration of CPR, respectively. Our findings were consistent with investigators arguing that asphyctic hypothermic patients underwent significantly longer periods of CPR, presented more frequently with asystole, had a more pronounced acidosis, and accordingly, higher levels of s-K^+^ (9, 28).

#### Survival and HOPE Survival Prediction Score

The present finding of a mean survival rate of 37% after ECLS rewarming is consistent with that reported by Pasquier et al. This is not surprising since 12 of the 20 studies included in their analysis overlapped with those enrolled here (20). The latter investigators suggested a HOPE survival prediction score, based on variables available on admission to hospital. According to ROC analysis, their score outperformed outcome prediction based solely on the serum potassium concentration, which hitherto had gained support as the single best predictor of survival from HCA. Pasquier et al. recently validated the score by studying a cohort of 122 patients with HCA out of whom 42% survived. This survival rate was not far from the predicted mean survival probability of 38%, which they calculated with the HOPE score algorithm. The latter score corresponded with a ROC curve with an AUC of 0.825 (CI 95% 0.753–0.897), confirming that their model had a good discrimination ability (137).

In the subset of patients from whom we could retrieve individual data, we found a survival rate of 38.5% and determined the predictive probability of surviving ac-cording to a mean HOPE score of nearly 34% with significant difference between survivors and non-survivors with medians of 58.5 and 6.0%, respectively. However, we found no significant differences in score between ECMO-treated and CPB-treated survivors and non-survivors, respectively. In our study, ROC curve analysis with comparison of the AUC values between the HOPE score and the serum potassium concentration, supported the suggestion (20, 137) that the HOPE score is a better predictor of survival as compared with serum potassium (*P* = 0.0426; Figures 3A–C).

#### Influence of s-K^+^, pH, and Lactate on HCA

Not surprisingly, decreasing pH correlated with increasing lactate and s-K^+^ concentrations, as shown in Table 4. Based on available literature, it has been hard to reach consensus on the concentration of s-K^+^, above which, survival appears futile and attempts on resuscitation should be discouraged. Farstad et al. observed that asphyctic patients were exposed to longer duration of CPR, higher s-K^+^ levels, more pronounced acidosis and presented more frequently with asystole as compared to non-asphyctic individuals (9). Until a few years ago, a s-K^+^ concentration >6.0 mmol/L was considered as a contraindication to ECLS after drowning and avalanche accidents (138). Nevertheless, Locher et al. (10) successfully rewarmed an avalanche victim with a serum potassium of 6.4 mmol/L nearly 30 years ago, and more recently experts on mountain medicine suggested s-potassium cut-off of 7 mmol/L in avalanche victims (139). According to the American Heart Association Guidelines for Cardiopulmonary Resuscitation and Emergency Cardiovascular Care, the upper survivable limit of s-K^+^ is unsettled for avalanche victims and hypothermic children (140) whereas the European Resuscitation Council Guidelines for Resuscitation of 2010 state that avalanche victims are not likely to survive if they are buried initially and in cardiac arrest on extrication with an initial s-K^+^ > 12 mmol/L (141). These authors based their recommendation on the reasoning that the highest values ever measured in survivors of drowning and exposure to extreme cold were 11.3 and 11.8 mmol/L, respectively (59, 99). Consequently, Brown et al. suggests a limit for s-K^+^ of 12 mmol/L for other causes of HCA than avalanche burials (142) whereas other experts discourage rewarming with ECLS for avalanche victims or stiffly frozen and traumatized patients of HCA with s-K^+^ > 12 mmol/l (143).

A pH of 6.29, recorded in a 6-year-old boy after near drowning, appears to be the lowest pH ever measured in a survivor of HCA. His initial body temperature was 17°C and his s-K^+^ peaked at 6.68 mmol/L. He underwent resuscitation with CPB after nearly 2 h of CPR and survived with diffuse cerebral atrophy (15). Two near-drowned patients were admitted to hospital with a pH of 6.33 and rectal temperatures of 36.7 and 33.5°C, respectively, after primary successful resuscitation (144). The first, a young man suffering from alcohol abuse and liver cirrhosis died after 22 h. The second, a previously healthy man, 24 years of age, left hospital 8 days later and resumed his prior occupation. The rectal temperatures might explain the large fall in pH despite that the submersions were assumed to be of <10 min duration. Most likely, the patients had suffered from asphyxia before the body temperature and the metabolism had fallen to a level that balanced the oxygen supply during manually performed CPR. The authors suggest that these patients produced lactic acid from excess pyruvate instead of generating ATP (144).

#### Influence of Coagulation and Fibrinolysis

Victims of HCA, particularly those with a history of trauma, present with derangement of the coagulation system. However, there is no consensus as to whether these changes are due to asphyxia, acidosis or hypothermia *per se*, or to a combination. Mitrophanov et al. recently observed that thrombin generation can be noticeably impaired even in moderate hypothermia (145). Boue et al. found that excessive activation of the coagulation system, as part of the post-cardiac arrest syndrome, accounts for early mortality of avalanche victims and suggested that global exploration of blood coagulation by means of thromboelastography or thromboelastometry should be mandatory in AH patients (37). Other investigators believe that activation of the coagulation system concomitant with a down-regulation of the fibrinolytic system contribute to the development of multiple organ failure in patients with post cardiac arrest syndrome (146). Wolberg et al. argue that coagulation examination, which is easily accessible on hospital admission, should be used to help making the decision of whether to prolong resuscitation (147). According to Debaty et al. assessment of coagulation should be used, in addition to other prognostic factors, when making decisions as to whether or not to prolong resuscitation from HCA (39). In our opinion, every relevant information should be taken into account for reestablishing homeostasis in the coagulation and fibrinolytic systems when rewarming patients with HCA.

#### Recent Advancements in Resuscitation From HCA

Morita et al. observed that rewarming of 38 victims of AH from the site of accident, with PPCPB, a portable version of ECMO, was associated with 84.2% survival as compared to 46.7% survival in 30 conventionally rewarmed patients. In the PPCPB group, five of six victims of HCA (83.3%) survived vs. one of 7 (14.3%) in the conventionally rewarmed group (3). Correspondingly, Ohbe et al. noted that in patients with accidental hypothermia without vital signs, resuscitation with VA-ECMO was associated with higher survival and more favorable neurological outcomes compared with conventional CPR alone (4).

Impressively, Wanscher et al. reported successful rescue operations followed by rewarming with ECMO from primary drowning of 5 male and 2 female high school students (32). The victims were floating lifeless with their faces submerged in salt water of 2°C after their dragon boat had capsized. After rescue, CPR started immediately. The first patient was admitted to a local hospital, where an arriving ambulance helicopter team resuscitated him with a portable ECMO system. He was weaned off after cardiopulmonary stabilization, 4 h later. The other 6 patients underwent resuscitation with ECMO at two different tertiary hospitals. Three boys and 2 girls recovered with good outcome while 2 boys survived with severe sequelae. Six females and 1 male student involved in the same accident rescued themselves by swimming ashore (32).

Sawamoto et al. reported successful rewarming of 26 subsequent patients of HCA of various etiologies applying a portable version of ECMO. Ten patients recovered fully while 16 (62 %) had sequelae (38). This indicates that portable ECMO might play a potential future role in the resuscitation of patients of HCA directly from the scene of accident.

#### Case Reports

This review and meta-analysis presents more individual case reports than any previous systematic review we know of. Although anecdotal, case reports describe novel or unusual therapeutic options of treating rare conditions (148), as surveyed in Figure 5 and Table A5. Case reports may also elucidate the potential benefits of hypothermia as a means of protecting the brain against hypoxic damage, for instance, during circulatory arrest for neurosurgery or cardiac surgery in children (149). Vretenar et al. summarized 68 case reports in a collective review of severely hypothermic patients whereof 72% underwent rewarming with ECLS and 60% survived. When the authors excluded solitary cases in favor of case observation studies, survival fell to 50% (150). However, it should be borne in mind that even those who recover almost completely from long lasting HCA might suffer from small fiber neuropathy (151).

Most case reports present patients with successful outcomes who do not follow the expected fatal course of illness. Therefore, their scientific value is ambiguous (152). In order to avoid selection and publication bias, we did not include single case reports in the present meta-analysis of observational studies (153). Nevertheless, we cannot exclude the possibility that some of the case reports also have been included in the studies constituting the main cohort. More than 90% of the patients presented as case reports, and as many as 96% of those reported with rescue collapse or witnessed cardiac arrest, left hospital alive. Probably, most of them breathed spontaneously while their body temperatures decreased gradually until the inception of HCA. Thus, most of them were non-asphyctic. Surprisingly, the 41 years old man (70), who fell into the ditch obtained a HOPE survival prediction score of only 14% against 54% in the toddler whose rectal temperature fell to 11.8°C (123) and 87% in the female skier, who experienced a drop in rectal temperature to 13.7°C (Table A5) (82). The reason for this big difference in survival prediction, was mainly due to longer CPR time, male sex and a higher core temperature before rewarming with CPB could be initiated (70, 137).

## Limitations

Most obvious limitation of this study is that the included reports cover a period of nearly 4 decades. During this period, general knowledge of managing HCA has progressed along with technical improvements, including the introduction of ECMO with heparin-coated membranes for rewarming victims of HCA (6). The oldest reports with patients rewarmed with CPB tended to have very high s-K^+^ levels and low pH levels that could be suspicious of asphyxia and longer periods of cardiac arrest before CPR was started. High s-K^+^ levels and low pH levels also were significantly associated with increased odds of not surviving HCA, independent of ECLS technique (Table A3). In the subset of patients from whom we had individual data, s- potassium was significantly higher and pH was significantly lower in the CPB group as compared with the ECMO group. However, by removing 10 patients with s-K^+^ ≥ 11.8 mmol/L, these differences vanished.

The present review and meta-analysis may not be free of confounding. As for most observational, non-randomized retrospective studies, the data originates from small patient populations with a variety of etiologies of AH and a diversity of co-morbidities. We intended to control for risk factors by adjusting for them in logistic regression analyses, but unfortunately, individual data were not accessible in all the studies of the main cohort. Since the data were partly parametrically and partly non-parametrically distributed, they could not be compared in a meta-analysis between survivors and non-survivors and between subjects undergoing the two different rewarming techniques. By introducing a subset of studies with individual data, we were able to compensate for some of these disadvantages, but admittedly, several variables were underpowered for these comparisons.

We cannot exclude the possibility that publication bias is another factor reducing the trustworthiness of the meta-analysis. Looking more closely at the data of the studies included in the main cohort, it appears that only the report of Ruttmann et al. had the necessary power to compare rewarming with CPB and ECMO in the same study (8). A uniform system for reporting like the Utstein style guidelines for reporting of drowning (154) is missed for patients with HCA. Of understandable reasons, focus on reporting details, such as correct notification of initial rectal or core temperature, will easily be neglected when lifesaving procedures have the highest priorities.

Possibly, the higher risk for not surviving attempted rewarming from HCA for a man as compared to a woman, might be due to different risk-behavior between the sexes. As a matter of fact, the subset displaying individual data revealed that almost 5 times as many men as women had HCA after avalanches and 3 and 2 times, respectively, as many male adults and children drowned as compared with adult women. The present study revealed that men also were overrepresented in the group of victims (71 vs. 29% women) undergoing HCA after exposure to cold such as fall into crevasse, water immersion or falling asleep outdoors after intoxication. This support the assumption that an inherited female trait cannot be excluded (131).

## Summary

We present 23 case observation studies in a meta-analysis aimed at comparing outcomes of rewarming of victims of HCA by means of CPB or ECMO with CPB or ECMO. Overall, independent of resuscitation technique, the probability of surviving was 50% higher after witnessed as compared to unwitnessed HCA. Moreover, there was a more than 40% greater probability of surviving after ECMO, as compared with CPB. Both ECLS techniques considered together, women had a 76% greater probability of surviving as compared with men. Correspondingly, the chance of surviving with ECMO and CPB of women were 67 and 86% greater, respectively, in comparison with men. We found higher risks of dying with higher body temperatures, higher values of potassium, sodium and PaCO_2_, and lower pH and PaO_2_. The probability of surviving with good vs. poor neurological outcome was 14 % higher with CPB as compared with ECMO whereas the chance of surviving with poor neurological outcome rather than dying was higher with ECMO than with CPB. The longer duration of cardiopulmonary support provided by the ECMO-treatment, made it possible for the latter patients to survive and leave hospital alive despite they had neurological deficits. In a subset of patients displaying individual data, mean predictive surviving probability (HOPE score) was 33.9 ± 33.6% with no significant difference between ECMO and CPB-treated patients. We also summarized 80 case reports with 96 abbreviated medical records of individual patients, albeit without including them in the meta-analysis. International guidelines for clinical management and reporting of outcomes of rewarming of victims of HCA are highly missed.

## Data Availability Statement

The original contributions generated for the study are included in the article/Supplementary Material, further inquiries can be directed to the corresponding author/s.

## Author Contributions

ES and TT took the initiative to an educational collaboration in managing severe accidental hypothermia between UiT, The Arctic University of Norway and the Postgraduate Institute of Anesthesiology and Intensive Care Medicine, North-Western State Medical University, named after I. I. Mechnikov, St. Petersburg and the Department of Anesthesiology and Intensive Care, Northern State Medical University, Arkhangelsk, The Russian Federation. The co-workers agreed on writing a review and meta-analysis on resuscitation from hypothermic cardiac arrest by means of ECLS. We designed the study in two meetings, the first in Tromsø and the second in St. Petersburg. LB suggested the topic, drafted the manuscript and interpreted data together with KH, who was responsible for the statistical analysis. KH also contributed to the study design, critically appraised the conclusions drawn, and revised the manuscript. TN contributed with valuable suggestions concerning the study design. ER performed and up-dated the literature searches, as outlined in Methods, contributed with figures, and revised the manuscript. TN, ER, ES, MK, TT, and KL made critical contributions to the study. All authors read and approved the final manuscript.

## Conflict of Interest

The authors declare that the research was conducted in the absence of any commercial or financial relationships that could be construed as a potential conflict of interest.

## Abbreviations

AH: Accidental hypothermia
ATP: Adenosine triphosphate
AUC: Area under the curve
o C: Degree(s) Celcius
CA: Cardiac arrest
CI: Confidence interval
CO: Cardiac output
CPB: Cardiopulmonary bypass
CPC: Glasgow-Pittsburgh Cerebral Performance Categories (CPC) scale
CPR: Cardiopulmonary resuscitation
ECLS: Extracorporeal life support
ECG: Electrocardiogram
ECMO: Extracorporeal membrane oxygenation
EEG: Electroencephalogram
GPS: Global positioning system
HCA: Hypothermic cardiac arrest
HOPE score: Hypothermia outcome prediction after extracorporeal life sup-port score
hr(s): Hour(s)
ICU: Intensive Care Unit
IQR: Inter quartile range
Min(s): Minute(s)
OHCA: Out of hospital cardiac arrest
OR: Odds ratio
PaCO_2_: Arterial partial pressure of arterial carbon dioxide
PaO_2_: Arterial partial pressure of oxygen
PaO_2_/FiO_2_: Ratio of arterial oxygen tension to inspired gas oxygen fraction
PEEP: Positive end-expiratory pressure
PPCPB: Portable percutaneous cardiopulmonary bypass system
PRISMA: Preferred Reporting Items for Systematic Reviews and Meta-Analyses
PROSPERO: International Prospective Register of Systematic Reviews
RR: Relative risk ratio
RCT: Randomized controlled trial
ROC: Receiver operating characteristics
ROSC: Return of spontaneous cardiac activity
SD: Standard deviation
s-K^+^: Serum potassium
s-Na: Serum sodium
VA: Veno-arterial.
