# Supplementary material for: Rewarming From Hypothermic Cardiac Arrest Applying Extracorporeal Life Support: A Systematic Review and Meta-Analysis
Source: Front Med (Lausanne). 2021 May 13;8:641633. doi: 10.3389/fmed.2021.641633 (PMC8155640; doi:10.3389/fmed.2021.641633)
Supplement: Supplementary file 1 [file Data_Sheet_1.pdf]

Appendix

Table 1A | Incident causing hypothermic cardiac arrest (HCA), duration of cardiopulmonary resuscitation (CPR) and ECG in victims of hypothermic cardiac arrest (HCA). Electrocardiography (ECG) changes: asystole, ventricular fibrillation VF), atrial-ventricular (AV) block or pulse-less electrical activity (PEA) and lung edema or ARDS reported in observational studies.

	**Incident Causing HCA**				**Duration of CPR (hrs)**		**ECG**			
**First author [reference number]**	**Ava-lanche (n)**	**Submersion (n)**	**Exposure. immersion or crevasse (n)**	**Witnessed or rescue collapse (n)**	**Survivors (n)**	**Non-Survivors (n)**	**Asystole (n)**	**VF (n)**	**AV block. PEA, other causes (n)**	**Lung edema or ARDS (n)**
Splittgerber [26]			6	1	1(r0.58-1)	1.5(r4.5-6)	2	2		5
Brunette [27]		1	9	5			2			
Mair [28]	12	7	3		2.25(r0.67-3.5)	2.75 (r0.67-6)	13	9		5
Letsou [29]		3	2	3			1	4		
Locher [10]* &Walpoth [30]	1		14	9	2.35±0.83		5	10		11
Hauty [31]							9		2	
Ruttmann I [8]	22	22	15	10			31	25	3	9
Wanscher [32]		7		2	1.08(r0.93-2.08)		2	4	1	
Silfvast [33]		3	20	4	1.08(IQR0.73-[illegible]	1.17(IQR1.04-[illegible]	7	15	1	
Morita [3]							4	1	1	
Schober [34]			8	6	3.1(r0.16-5.25)	3.75(r1.67-9.3)	4	3	2	
Weuster [35]		7	1		1.1	2(r1-2.3)				
Moroder [36]	4			5			2	1	1	
Boue [37]	19			6	0.22(r0.8-0.92)	1.58(r0.17-5.00)	14		6	
Sawamoto [38]	2	12	12		1.13(IQR1.06-1.4)		16	10		
Debaty [39]	6	4	13	13	1.08 (r0.33-4.41)	2(r 0.17-4.00)				
Champigneulle [40]		20			0.6 (IQR0.5-0.7)	0.7(IQR0.6-1.0)	20			
Hilmo [12]	8	21	6	8	3.06(r1.42-4.12)	2.47(r1.8-7.83)	22	6	7	10
Darocha [41]	2	1	7		2.5(r1.8-5.8)	4.2(r2.6-4.7)	5	5		
Svendsen [42]		49	19		1.58 (r0-3.25)	2.5(r0.75-4.97)				
Khorsandi [43]		1	9		3.25(r2.83-4.16)	2.36(r2.16-3.26)	3	5	2	3
Ruttmann II [44]	19	18	14	14				16		

ECG, electrocardiography; IQR, interquartile range; r, range; n, number of patients

Table 2A | Serum potassium (s-K+), s-pH and s-lactate in survivors and non-survivors of hypothermic cardiac arrest, who underwent attempted rewarming with ECLS (CPB or ECMO).

First author [reference number]	s-K^+ (mmol/L)		pH		Lactate (mmol/L)	
	Survivors	Non-survivors	Survivors	Non-survivors	Survivors	Non-survivors
Splittgerber [26]	4(r2.5-4)	4(r4-4)	7.29(r6.97-7.42)	7.78(r7.3-8.26)		
Brunette [27]	4.05(r3.8-9)	4.1(r2.8-5.4)				
Mair [28]	4(r3.8-4.1)	6.5(r3.4->20)	7.09	6.7(r6.5-7.1)		
Letsou [29]	3.15(r2.5-3.8)	2.9(r2.6-3.2)	6.9(r6.7-7)	7.1(r7-7.3)		
Locher [10]* &Walpoth [30]						
Hauty [31]	6(r3.1-9)	22(r8-33)	7.23(r7.45-7.0)	6.89(r6.88-7.96)		
Ruttmann I [8]	4.9±1.9	8.1±4.3	6.80±0.32	6.64±0.24	13.11±6.55	15.20±7.13
Wanscher [32]	3.9(r2.3-4.3)		6.61(r6.43-6.94)		21.0(r9.4-24)	
Silfvast [33]	3.6(r3.1-4.1)	5.0(r3.8-6.6)	6.92(r6.77-7.05)	6.66(r6.58-6.80)		
Morita [3]						
Schober [34]	3.1(r2.9-4)	4.55(r1.4-8.2)	7.26(r7.19-7.29)	7.08(r6.93-7.36)	9.8(r6.1-10.9)	6(r2.7-9.5)
Weuster [35]	5	5.59(r3.5-8.54)	6.8	6.97(r6.8-7.18)	15	15(r9-20)
Moroder [36]	4.6(r3.8-6.6)		6.58(r6.5-6.9)		19.8(r10-26.8)	
Boue [37]	3.2(r2.5-4.2)	5.6(r2.8-10)	7.19(r7.2-7.3)	6.73(r6.25-7.25)	7(r5.26-11.9)	14.2(r6.9–29.1)
Sawamoto [38]	4.7(IQR3.8-6.3)		6.95(IQR6.78-7.22)		13.4(IQR9.4-24.3)	
Debaty [39]	3.2(r2.10-5.8)	4(r2.56-13.9)	7.16±0.23	6.84±0.30	5.5(r1.6-8.6)	14.1(r2.1-28)
Champigneulle [40]	4.8±0.4	5.9±2.4	6.77±0.19	6.79±0.27	19±9	19±7
Hilmo [12]	6.73±0.81	8.8±2.9	6.88(IQR6.7-7.47)	6.67(IQR6.39-6.84)		
Darocha [41]	3.7(r2-4.6)	5(r4.4-6.4)	6.91(r6.64-7.14)	6.67(r6.56-6.99)	12(r7.1-17)	14(r11.9-16)
Svendsen [42]	4.0(r2.2-7.9)	6.1(r2.5-25)	6.87(r6.36-7.23)	6.62(r6.23-7.21)		
Khorsandi [43]	3.8(r3.7-6.1)	3.2(r3.3-4.2)	7.01(r6.8-7.1)	6.79		
Ruttmann II [44]						

Data presented as mean ± standard deviation (SD), median and range (r) or median and interquartile range (IQR)

Table 3A| Physiological variables in survivors and non-survivors from a subset of studies of accidental hypothermic cardiac arrest.

Physiological variables	Survivors	n	Non-survivors	N	P-value
Initial temperature °C, mean ± SD	22.8°C ± 3.7°C	76	24.6°C ± 3.72°C	123	0.0016
pH, mean ± SD	6.96 ± 0.26	61	6.77 ± 0.03	108	0.001
K^+ mmol/L, median (IQR)	3.8 (1.3)	64	5.6 (4.2)	113	<0.001
$PaCO_2$, kPa, median (IQR)	7.5 (3.70)	21	9.6 (10.65)	73	0.0018
PaO_2, kPa, median (IQR)	15.95 (40.05)	20	11.40 (19.30)	71	0.039

n, number of observations; SD, standard deviation, IQR, interquartile range

Table 4A | Odds ratios of different variables from univariate logistic regression with death as outcome after attempted rewarming by means of ECLS (CPB or ECMO).

Variable	All patients				CPB			ECMO		
	n	SD	OR	P-value	n	OR	P-value	n	OR	P-value
Age*	199	20.4	0.71	0.0237	106	0.70	0.0916	93	0.74	0.1726
Sex male	200		3.58	0.0002	107	3.28	0.0132	93	3.74	0.0063
Temp initial	199	3.8	1.61	0.0023	106	1.35	0.2059	93	1.84	0.0043
Sodium*	87	9.2	1.83	0.0301	27	1.01	0.9760	60	3.64	0.0024
Lactate*	80	6.6	1.90	0.0192	8	1	1	72	1.74	0.0427
pH*	169	0.3	0.48	0.00009	87	0.45	0.0039	82	0.55	0.0180
PCO_2*	104	6.2	2.09	0.0111	52	1.61	0.1951	52	3.11	0.0326
PaO_2*	101	21.5	0.56	0.0080	49	0.46	0.0520	52	0.61	0.0708
K^+ initial*	177	3.3	5.53	0.000002	92	2.75	0.0074	85	23.2	0.0001
CPR duration*	145	92.9	1.72	0.0116	60	2.33	0.0309	85	1.37	0.2221

*indicate OR is per 1 standard deviation increase of variable, while the rest are per unit increase.
n, number of observation; HCA, hypothermic cardiac arrest

Table 5A | Eighty case reports presenting (abbreviated) the medical records of 96 patients with hypothermic cardiac arrest (HCA), who underwent rewarming using ECLS (CPB and / or ECMO).

First author; (year); [reference number]	Male (M); fem-ale (F) age (years)	Avalanche (A); crevasse (C); immersion (I); submersion (S); trauma (T); urban (U)	Witnessed HCA: yes/no; lowest core temperature (º C)	Duration of HCA or CPR (min). ECG: ventricular fibrillation, VF; asystole, asyst; SR, sinus rhythm	pH; K+ (mmol/L); lactate (mmol/L)	CPB or ECMO; sternal (ST) or Veno-arterial (VA) femoral access; time (min. hrs or days)	Lung edema/ Pneumonia /Lung congestion	Time to ICU or hospital discharge (days)	Condition at discharge; sequela
Fell R (1968) [2]	F/42	I; barbiturate intoxication	Yes; 22 º C	CPR for 210 min; defibrillated at 34 º C	pH 7.86	VA femoral CPB;	Lung edema; pneumonia; empyema;	Hospital discharge after 35 days	No sequela; working part-time
Towne W (1972) [45]	M/58	U; alcohol intoxication	Yes; 25 º C	CPR for 120 min	pH 8.80	VA femoral CPB for 45 min	Pneumonia. Acute respiratory failure	57 days in hospital	Amputated left leg; intellectually impaired.
Truscott D (1973) [46]	F/23	U; found alcohol-influenced after 14 hrs in snow beside her car.	Yes; 22 º C. VF in Emergency Dept.	CPR time lacking	Lacking data	VA femoral CPB. Rewarmed to 37 ºC. Successfully defibrillated	Lung complications requiring 1 month of mechanical ventilation	Spinal paraplegia due to C2 fracture after car accident	Fully awake until she died unexpected 4 months after the accident.
Wickström P (1976) [47]	F/65	U; found in abandoned building.	No; 25 º C	CPR for 60 min. VF; attempts on defibrillation	pH 7.31; $PaCO_2$ 59 mm Hg; K+ 6.95 mmol/L	VA femoral CPB for 85 min	No	Rewarmed to 33 °C, but unsuccessful defibrillation.	Unsuccessful resuscitation. Died
Wickström P (1976) [47]	M/64	U; found on sidewalk outside his home.	No; 23.8 º C	CPR for > 120 min; intubated on spot.	pH 6.95; $PaCO_2$ 50 mm Hg; K+ 5.6 mmol/L	VA femoral CPB for 100 min	No. but pleural effusion due to metastasis from adenocarcinoma.	Necessitated vigorous water and potassium replacement.	Neurologically recovered. Trans-metatarsal amputation of one foot.
Wickström P (1976) [47]	F/46	U; found on snowbank with VF.	Yes;<20 º C	CPR for >60 min after arrival at hospital	pH 7.83 after HCO_3^- 70 mmol $PaCO_2$ 38	VA femoral CPB for 50 min	Yes; Pneumonia treated with antibiotics and bronco-alveolar lavage	Successfully defibrillated	Discharged from hospital with no sequela
Althaus U (1982) [48]	M/42	C; 300 min	No; 19 º C	CPR for 165 min	Lacking data	VA femoral CPB	No	Lacking data	Fully recovered
Althaus U (1982) [48]	F/24	C; 150 min	Yes; 24 º C	CPR for 165 min	Lacking data	VA femoral CPB	No	Lacking data	Fully recovered
Bjertnæs L (1987) [49]	M/3	S; run from nursery; found lifeless drowned in the Norwegian Sea	No; 26 º C	CPR during 120 km helicopter transfer to local hospital and attempted	pH 6.96	CPB via ST. VF, defibrillated to SR. ROSC 7 hrs after incident. Moved his	Yes. 20 min after weaning from CPB, Mean BP transiently 90 mmHg. Suddenly, alveolar flooding and pupillary dilation. CPB restarted.	After restart CPB, unsuccessful resuscitation. CPR terminated in the operating room	Died with MOF and signs of general capillary leaks.

				rewarming. Further transport 322 km to UH.		head; both pupillae constricted.			
Feiss P (1987) [50]	F/27	U; found comatose in wet field at a ambient temperature of 4 °C	Yes; 19 °C	CPR (?). VF upon arrival to emergency room.		VA femoral CPB for 63 min. Return of VF, which was defibrillated at 33 °C	Yes. transient lung edema	Successful resuscitation.	Recovered completely. Discharged after one week in hospital.
Bolte R (1988) [79]	F/2.5	S; in creek for at least 62 min	Yes; 19 °C	CPR for 180 min. VF converted spontaneously to SR at 25 °C.	pH 7.25	VA femoral ECMO	Yes. severe lung edema; mechanical ventilation for 6 days	Awake after 3 weeks; discharged after 8 weeks.	Initially, cortical blindness. No sequela after 1 year.
Laub G (1989) [51]	F/60	U; obese patient found unconscious in cold (1 °C) room.	No; 26 °C	CPR including tracheal intub-ation for 20 min	Lacking data	VA femoral CPB for 90 min.	No; weaned from all pressors and regained consciousness after 72 hrs.	Recovered within 3 days. Discharged from hospital after 7 days.	No sequela
Graf D (1989) [52]	M/2.7	S; found after 45-60 min in neighbor's pond.	No; 20 ° C.	CPR for approximately 240 min	6.7; CPK 10072 U/L; Myoglobin 422785 µg/L	CPB via ST for 1 h. Dis-connected at 36 °C after obtaining SR	No; rhabdomyolysis and DIC	1 1/2 days in ICU; No EEG activity.	Died in ICU
Graf D (1989) [52]	M/3.5	S; found after 15-20 min in neighbor's pond.	No; 18.4 ° C.	CPR for approximately 225 min	7.06; CPK 16430 U/L; Myoglobin 8120 µg/L	CPB via ST for 2 hrs until body temp of 37 °C after obtaining SR	Yes; lung edema; aspiration pneumonia	Discharged from ICU after 14 weeks	Completely recovered
Husby P (1990) [53]	M/51	I/S; Alcohol intoxicated fisherman; I/S for 40 min.	No; 24 ° C	CPR for 190 min before start of CPB; ECG converted to SR at 31.5 °C	pH 6.97	VA femoral CPB support 60 min post ROSC	Yes; cardiac failure	Lung edema; extubated after 24 hrs in ICU. Discharged after 12 days;	No neurological sequelae 6 months later
Black J (1992) [54]	M/58	U; alcolintox; in police custody	No; 29 ° C	After CPR for 45 min. transfer to center for cardiac surgery.	Lacking data	VA femoral CPB for 103 min. VF converted to SR at 30 °C.	Yes; bilateral pneumonia.	Tracheostomy and stay in ICU because of mechanical ventilation.	Discharged without neurological sequelae after 2 weeks
Wong P (1992) [55]	M/middle aged	U; collapsed outdoors	yes; 24 ° C	After CPR for 90 min. transfer to center for cardiac surgery	Lacking data	VA femoral CPB for 180 min. VF converted to SR at 29 °C.	Yes; cardiac failure; inotropic support for 24 hrs	Pronounced brain stem "dead" after 48 hrs in ICU.	Died in ICU
Bolgiano E (1992) [56]	M/22	U; found unconscious in park due to alcohol intoxication and head injury.	Yes. 24 ° C. upon loading into ambulance. ECG changed to VF	CPR for 60 min during transfer to center for cardiac surgery and preparation for CPB	pH 7.18; K^+ 5.8	VA femoral CPB for 50 min; VF converted to SR at 30 °C.	Cerebral contusion on CT caput and development of ARDS on 5th day.	Only responsive to pain until he was discharged to a nursing home after 63 days.	After 1 year: light cognitive impairment and peripheral polyneuropathy.

Cha SO (1992) [57]	F/37	U; depression and alcoholin-toxication. Found lifeless in a remote forest after 10. 5 hrs. Air temperature 0 ° C	Yes. 23.2 ° C. ECG: PEA alternating with nodal rhythm and periods with VF	CPR for 200 min during transfer to center for cardiac surgery for CPB	pH 7.17; K+2.7	ST CPB for 67 min.	No; mental confusion the first postoperative days	Totally, 10 days in hospital	Discharged home after 10 days. No neurological sequelae one year later.
Cha SO (1992) [57]	M/42	U; depression; barbiturate- and alcohol intoxication. Found lifeless in his car after 12 hrs. Air temp +1 ° C	Yes; 29.5 ° C rectal. Initially SR. but no pulses.	CPR until start CPB	pH, K+ within normal limits	Femoral VA CPB for 80 min.	No; complications	He suffered from tetraplegia after a previous trauma	Discharged home in his previous condition. No neurological sequelae. due to AH
Norberg W (1992)[75]	M/11	S; fell through crack in the ice; submersed for 45 minutes	No; 24 rectal with nadir of 22 °C during CPB. ECG initially asyst-ole.	CPR for130 min and defibrillated 38 times before start ECMO	pH 6.72; K+ 3.4	VA femoral CPB from 61 min after hospital admission. Changed to median sternal CPB. ROSC after 20 min.	Yes, fulminant edema treated with assisted ventilation with positive end-expiratory pressure of 15 cm H2O. Furosemid, CaCl2 and dopamine infusions.	Initially cold-induced peripheral muscle weakness. Weaned from mechanical ventilation after 5 days. Day 18, no signs of central neurological injury.	Fully recovered. No deficits found at follow-ups during the subsequent two years.
Antretter H (1994) [58]	M/6	S; fell into ice-cold mountain stream; found lifeless 6.5 km downstream 65 min later.	No; 16.4 ° C	Asystole from site of accident. CPR for 160 min	pH 6.51; K+ 7.6 mmol/L	VA femoral CPB for 96 min. At 32.9 ° C, VF. which was defibrillated to SR.	Extubated on day 6 in ICU; rhabdomyolysis due to skeletal muscle hypoxia.	In ICU, cold-induced peripheral neuropathy; no heart -or lung damage.	Ten months after the accident, the polyneuropathy had vanished.
Waters D (1994) [77]	M/31	I/S for > 1 h after snowmobile accident on frozen lake	No; 23 ° C	CPR for 66 min Tracheal intubation at the site of accident	pH 7.26	VA femoral ECMO. weaned off after 180 min	Yes. ARDS	Extubated after 12 days of mechanical ventilation	Discharged from hospital after 17 days with bilateral brachial plexus neuropathy
Tyndal C (1996) [78]	M/21	U; alcohol intoxicated driver in car accident	No; 27 ° C	CPR for 93 min	pH 7.02	VA femoral ECMO. Blood pressure 92/62 mmHg 23 min later	Yes. lung edema	Weaned from ECMO after 2 hours and 12 min. Chest tube because of pneumothorax	No major complication. Discharged from hospital after 4 days.

Dobson J (1996)[59]	F/2.6	U; left home; was trapped outdoors in conditions equivalent to - 42 ^{o}C	No; 14.2 ° C	CPR for > 100 min	pH 6.96; K+11.8	CPB via ST. Disconnected after 4 h 56 min	Yes. Pneumonia	ICU and hospital discharge after 15 and 30 days, respectively	Amputation of gangrenous left leg; transiently small muscle weakness
Steedman D (1997) [60]	M/29	I; fell through the ice while walking his dog. Rescued and awake after 80 min	Yes; 28.3 ° C	CA in ambulance. CPR for 143 min until start CPB	pH 7.1; K^+ 6.1	CPB via ST	No; weaned off CPB with SR after 78 min.	In ICU for 72 hrs ;artificial ventilation and inotropic support	No neurological deficit.
Kumle B (1997) [115]	M/21	S; Submersed for 30 min. Found on riverside with CA	Yes; 26.1 ° C	CPR for150 min	Lacking data	VA ECMO for 3 days.	Yes. severe DIC and ARDS	In ICU for 22 days. Left hospital after 41 days.	Peripheral muscular dysfunction.
Mair P 1997 [80]	M/54	C; Found 50 min after fall into narrow crevasse.	Yes; 23 ^{o}C; CA after difficult extrication; VF on ECG.	CPR for 90 min; attempts on defibrillation failed.	pH 6.7; K+ 4.1	VA femoral heparin-coated ECMO	No. ECMO disconnected after 35 min at a core temperature. of 34 ^{o}C.	In ICU for 3 days. Extubated after 48 hrs.	No neurological sequela. Left hospital 10 days later.
Irone M (1998) [81]	F/22	U; found outside after intoxication due to attempted suicide.	Yes; 24. 5 ° C. VF following bladder catheterization.	CPR began immediately after start of VF.	pH 7.18; K^+ 2.4;	VA femoral ECMO	Weaned from ECMO after 80 min	In ICU for 1 day. Then, weaned from mechanical ventilation.	Discharged from hospital the third day
Gilbert M 2000 [82]	F/29	I; A female skier; rescued with CA 79 min after she fell into glacier crack and was trapped in head down position, ice-cold water rinsing her face.	Yes; 13.7 ° C; isoelectric ECG	CPR for >130 min	pH 6.54; K+ 4.3 mmol/L initially, peaking at 8.2 mmol/L during CPB	CPB via ST for 179 min followed by femoral VA ECMO for 5 days	Yes. cardiopulmonary insufficiency and ARDS	ICU 28 days due to Critical Illness Polyneuropathy. Transferred to local hospital ICU after totally 35 days on mechanical ventilation	At 5 months residual paresis in extremities. Nearly full recovery 2 years later. Resumed her prior activities, both as a medical doctor, who specialized in radiology and as a skier.
Thalmann M (2001) [61]	F/3	S; Submersion	No; 18.4 ° C	CPR for 90 min	pH 6.72; K^+ 5.7	VA femoral CPB 6h	Yes; pulmonary edema. treated with ECMO for 15 hours	12 days in ICU	No sequela after 20 months
Wollenek G 2002 [15]	F/4	S > 15 min; found face down in pool	No; 22 ° C	CPR through open chest for nearly 60 min	pH 6.58; K^+ 5.8; lactat 21	CPB via ST for 141 min	Yes; cardiorespiratory instability	Chest open for 6 days. Therapeutic hypothermia and sedation until extubation after 2 weeks in ICU	Fully recovered 1 year later.

Wollenek G 2002 [15]	M/6	S; pushed into river by his mother	No; 17 ° C	CPR for 120 min	pH 6.29; K^+ 6.68	VA femoral ECMO for 438 min	Cardiopulmonary impairment		Diffuse cerebral atrophy
Wollenek G 2002 [15]	F/8	S; found in water	No; 24 ° C		pH 6.34; K^+ 6.2	ST; CPB for 232 min	No ROSC	Not in ICU	Died
Mulpur A (2004) [62]	F/20	U; heroin addict. Found with cardiac arrest on graveyard.	No; 25 ° C	CPR for 90 min from the cemetery. Tracheally intubated and defibrillated with no success	pH 6.59; K^+ 5.6	CPB via ST; weaned off after 42 min.	Yes; pulmonary edema.	3 days in ICU. Extubated after 36 hours.	Discharged from hospital without neurological sequelae after 15 days.
Brat R (2004) [63]	M/26	U; carbamazepine intoxication; attempt on suicide; found unconscious in snow at 0 °C	No; 19 ° C	Lacking information	pH 7.09; K^+ 4.0	VA femoral CPB not heparin coated for 158 min	No cardiopulmonary complications	5 days ICU	Discharged fully recovered after 3 weeks
Moser B (2005) [83]	M/62	U; found unconscious in snow-bank. Asystole occurred during landing at the hospital.	Yes; 21 ° C	CPR for nearly 30 min	Lacking data	VA femoral ECMO for 24 h	Aspiration pneumonia	Extubated after 16 days in ICU; discharged to rehabilitation after 27 days	Amputation of both hands and toes due to severe frostbite
Marquis C (2005) [64]	F/52	I/S; found after 15-20 min in cold river	No; 22 ° C	CPR for nearly 120 min	pH 7.00; K+ 6	VA CPB. Weaned after achieving SR at 36 ° C	Yes; bilateral edema. Aspirated 4 L from airways over 2 hrs	Spent 21 days in ICU because of ARDS.	Discharged from hospital without neurological sequelae.
Tiruvoipati R (2005) [84]	M/55	U; found on the roadside	Yes; 24 ° C	CPR for 140 min	pH 6.73; K^+ 6.4	VV ECMO between right internal jugular - and left femoral vein for rewarming	No; weaned off ECMO after 24 hours and off mechanical ventilation 24 hrs later	2 days in ICU	Recovered completely with no sequela

Eich C (2005) [85]	F/3	S; found after 20 min of submersion	No; asystole; 27.2 ° C	CPR for 140 min	pH 6.62; K^+ 3.7	CPB for 7 hrs followed by ECMO	Yes; weaned off ECMO after 4 days.	26 days in ICU. Extubated after 12 days.	Minimal neurological disorder including ataxia
Incagnoli P (2006) [107]	F/50	S; found with CA after jump from bridge across the river Seine	No; 22 ° C	CPR from site of accident to hospital	pH 7.0; K^+ 4.5 mmol/L; lactat 16 mmol/L	VA femoral ECMO	Yes; ARDS, pneumonia and renal failure; 360 min support on ECMO	21 days in ICU. 15 days of mechanical ventilation	Discharged from hospital with no neurological sequela
Hohlrieder M (2007) [86]	F/16	C; trapped in crevasse immersed in ice cold water	Yes; 22 ° C	CPR of unknown duration	Lacking data	VA femoral ECMO	No; weaned off ECMO after 24 hours	Extubated and discharged from ICU after 3 days and from hospital after 22 days	No sequela
Hohlrieder M (2007) [86]	M/46	U; found unconscious on river bank after suicide attempt with insulin injection	Yes; 24 ° C	CPR during transportation because of VF	Lacking data	VA femoral ECMO	No; weaned off ECMO after 24 hours	Discharged from ICU after 4 days. Referred to rehabilitation-on after 10 days.	Hypoxic encephalopathy.
Iyer A (2007) [87]	M/28	S; diving clothed in wet suit. Found on the bottom after 30 min.	No; 24 ° C	CPR and adrenaline from site of accident	pH 6.67; lactate 22.4	VA femoral ECMO	Yes; cardio-pulmonary failure and lower limb ischemia.	Leg amputation. Weaned off ECMO after 1 day. Dialysis and mechanical ventilation for 5 and 7 weeks. respectively	Transferred to rehabilitation after 11 weeks in hospital.
Maisch S (2007) [65]	M/2	S. 10 min	No; 24.3 ° C	CPR for 80 min	pH 6.98; lactate 14.9	CPB via ST for 3 hrs	ICU with mild hypothermia	ICU 6 days. Discharged from hospital after 2 months	Fully recovered
Cooper S (2008) [88]	M/82	U; Alzheimer's disease. Found outdoors at air temperature of 10 º C.	Yes; 25.5 ° C	CPR for approximately 60 min because of PEA and VF	pH 7.16 ;K+ 3.3; lactate 3.0	VA femoral ECMO starting 58 min after arrival	Tracheostomy; weaned off ECMO after 4.5 hrs.	Discharged to elderly home after 10 days	Recovered to his premorbid status.
Oberhammer R (2008) [66]	M/29	A; found after 100 min at 3m depth; air pocket	Yes; 21.7 ° C; VF upon intubation	CPR for 165 min. interrupted by 15 min helicopter flight	pH 6.87	VA femoral CPB	Yes. pulmonary edema; connected to VA ECMO for 3 days	Transferred to his home hospital after 14 days. Totally. 17 days in hospital	Fully recovered
Binnema R (2008) [67]	F/40	I; found after > 4 hours after car accident	Yes; 23 ° C; VF upon intubation	Lacking data	pH 6.74; lactate 13.4	CPB via sternotomy	Yes; ARDS	3 days in ICU and 4 days in award	No sequela on discharge

Guenther U (2009) [89]	M/19	S. 12 min	Yes; 32.7 ° C	CPR for 20 min	pH 7.07	Start VV ECMO for 8 days	Yes; pneumonia	Hospital discharge day 27	No sequela on discharge
Guenther U (2009)[89]	M/12	S. 15 min	Yes; 32 ° C	CPR for 25 min	pH 7.11	VV ECMO for 6 days	Yes; pulmonary edema	Extubated after 8 days. In ICU for 10 days	No sequela on discharge
Lund F(2009) [68]	M/48	S; found floating head down in 3.5°C seawater after his kayak had capsized	No; 20.6 ° C; asystole. recorded with patient's heart rate monitor watch	CPR for 120 min	pH 6.67	Femoral VA CPB; ROSC 3h 27 min after CA	Yes; cardiac failure/pulmonary edema. Thiopental against ICP rises above 35 mmHg	21 days in ICU due to critical illness polyneuropathy. delirium and pneumonia	Discharged to rehabilitation after 33 days. Cognitive functions normal 12 months later.
Talbot S (2010) [74]	M/38	U; park - -11 °C surroundings	Yes; 26 ° C; asystole on tracheal intubation;	CPR for 84 min until start CPB	Lacking data	CPB after sternotomy. ROSC after 2.5 hrs	No	15 days in hospital	Discharged to rehabilitation facility
Sansone F (2011) [90]	F/40	U; found in railroad car with cardiac arrest. Blood alcohol 3.15 g/L	No; 23 ° C	CPR for 169 min	pH 7.25; K^+ 3.5;lactate 7.1	VA femoral ECMO for 7 hours 20 min	No; weaned from ventilator and extubated after 6 hours	2 days in ICU Discharged from hospital after 5 days	No sequela.
Sansone F (2011) [90]	M/50	U; found in railroad car with cardiac arrest. Blood alcohol 3.5 g/L	No; 25 ° C	CPR for 125 min	pH 6.98; K^+ 4.3; lactate 9.5	VA femoral ECMO for 3 hours 53 min	No; defibrillated at 28 °C. Weaned from ventilator 12 hours later	2 days in ICU. Then, moved to the general ward. Discharged on day 5	No sequela.
Putzer G (2012) [106]	M/48	U; polytraumatized skier after falling 100 m in a mountain side	Yes; 20 ° C; VF upon tracheal intubation	CPR from site of accident to starting ECMO 80 min after arrival to the hospital.	Lacking data	Femoral VA ECMO. Cerebral O_2-monitoring with NIRS	CT showed costa fractures pneumomediastinum. Diaphragm and pericardium ruptures	Near-infrared spectroscopy. transesophageal Doppler and invasive pressure monitoring	Died after 4.5 hrs in hospital receiving massive blood transfusions
Hagiwara S (2011) [91]	F/30	U; found in forest after drug overdose	No; 20 ° C	CPR for 172 min before ECMO; rewarming by hemodialysis	Lacking data	VA femoral ECMO with no heat exchanger for 3 days	Severe thoracic trauma	22 days in hospital	Recovered except for a slight left-sided muscular weakness
Sawamoto K (2012) [69]	M/57	S; found floating in water.	No; 22 ° C	CPR for 90 min	pH 7.02; K^+5.6; lactate 12.1	VA femoral CPB for 3.5 hours	Yes; aspiration pneumonia	Discharged from hospital on day 32	Full recovery

Mark E (2012) [70]	M/41	I; alcohol intoxication. Fell into creek. Was found after > 60 min. Cardiac arrest on rescue	Yes; 25 ° C	CPR for 273 min during transportation to hospital and start CPB	pH 6.88; K^+ 5.9	VA femoral CPB for 139 min	Yes; pneumonia	In ICU for 4 days. Transferred to local hospital after 12 days.	Discharged with no neurological sequelae
De Canniere L 2013) [92]	F/22	S. 7-10 min; landed in sea with paraglider	Yes; 29 ° C	CPR for 30 min until start ECMO	pH 6.8	VA ECMO for 72 h	Yes; ARDS; cardiac failure; EF 13 % day 1.	In ICU for 12 days	Full recovery on discharge from hospital day 12
Morley D (2013) [93]	F/45	U; Down's syndrome	Yes; 25 ° C	CPR for 50 min	Lacking data	VA ECMO for 3 hrs and 45 min	No	Extubated after 3 days. Discharged after 3 months	No sequela; recovered to her premorbid condition
Norberg P (2014) [76]	M/42	U; found outdoors at air temperature of +1 ° C; pulseless, alcohol intoxicated and not responding	No; 22 ° C	CPR for 130 min; 38 defibrillations until start VA ECMO	pH 6.9; lactat 10 mmol/L. Electrolytes within normal limits	Defibrillated at core temperature of 30 ° C. Weaned off ECMO at 35 ºC	No	Initially. EF 45% of normal. but normalized within 3 weeks	Recovered completely; no sequela 3 months after discharge.
Boue Y (2014) [94]	M/17	A; extricated after 6 hrs; air pocket	Yes; 21.1 ° C; Rescue collapse. VF	CPR for 20 min until start ECMO	pH 7.26; K+ 4.0; lactate 4.6	VA ECMO	No;	In ICU for 4 days	No neurological sequela (CPC score 1).
Boue Y (2014) [94]	M/41	A; buried for 7 hrs as indicated by his GPS-watch monitor; air pocket	Yes; 23.3 ° C rectal; asystole	CPR for 50 min until start ECMO	pH 7.19; K+ 2.8 lactate 7.0	VA ECMO	No;	In ICU for 5 days	No neurological sequela (CPC score 1).
Boue Y (2014) [95]	F/57	U; got lost in snow storm	Yes; 16.9 ° C	CPR for 307 min until start ECMO	pH 7.25; K+ 5.8; lactate 5.7	VA ECMO; SR after 81 min	VA ECMO for 3 days due to cardiac failure.	In ICU for 55 days due to rhabdomyolysis treated with hemofiltration. Discharged after 3 weeks.	Slight cognitive impairement
Meyer M (2014) [71]	F/65	U; found on snow-covered river bank	No; 28 ° C asystole	Manual CPR for 288 min	6.94; K^+ 2.8	CPB for 3hrs and 52 min	Rhabdomyolysis. Lung edema. EF 45 %.	In ICU for 5 days followed by 8 weeks rehabilitation. Auto-extubation day 4.	Fully recovered except for slight memory deficit after 5 years
Jarosz A (2014) [97]	M/56	U	No; 25 ° C	CPR for 15 min	Lacking data	VA femoral ECMO for 23 h		8 days in ICU	No sequela

Jarosz A (2014) [97]	M/55	U	No;22.2 ° C	CPR for 140 min	Lacking data	VA femoral ECMO for 22 h		11 days in ICU	No sequela
Jarosz A (2014) [97]	F/48	S	No; 32 ° C	CPR for 107 min	Lacking data	Not weaned off VA femoral ECMO		3 days in ICU	Dead
Darocha T (2015) [96]	M/?	U; found outdoors in the early morning	Yes; ?° C	CPR for 150 min including external rewarming	pH 6.91; K^+ 2.5; lactate 7.9	After external rewarming, VA femoral ECMO for 24 h	Yes, aspiration pneumonia. Extubated day 8	Discharged from ICU after 9 days	No neurological or cardiac sequela
Ginty C (2015) [98]	M/?	U; surrounded by drug paraphernalia	Yes; 27 ° C VF	CPR for 200 min	6.89; K^+ 3.4; lactate 14.1	VA ECMO for 48 h	No	6 days in ICU; 15 days in hospital	Full recovery
Romlin B (2015) [99]	F/7	S; submersed 83 min after sliding down a cliff	No; 13.8 ° C; asystole	CPR for 64 min. Initially. ice blocking the upper airways	6.6; K^+11.3; lactate 22	CPB between right atrium and aorta 3 hrs followed by VA femoral ECMO	Weaned off ECMO 5th day.	26 days in ICU. Extubated day 10. 7 days in neurological ward.	Fully recovered after 15 months
Kosinski S (2016) [100]	F/25	A; extricated with Glasgow Coma Scale 11 after 2 hrs.	Yes; 16.9 ° C; VF	CPR for 405 min; converted to SR at 24.8 °C	Lacking data	VA ECMO for rewarming and circulatory stabilization over 91 hrs	Yes;	6 days in ICU.	26 days in hospital. Full recovery 1 year after hospital discharge
Niehaus M (2016) [101]	F/25	U; Intoxication GCS 7 when found	Yes; 26.0 ° C; rescue collapse; VF upon intubation	CPR for 37 min. Connected to VA ECMO. Spontaneous SR at 36 °C	Lacking data	VA ECMO for 10 hrs	No;	37 days in hospital; suffered from ostbite. bilateral compartment ndrome and rhabdomyolysis	Amputation of metatarsi laterally. No neurological equela
Niehaus M (2016) [101]	M/26	U; Intoxication GCS 3 found in snowbank	No; 21.7 °C; asystole;	CPR for 180 min. VA ECMO. VF at 28.8 o C converted to SR	Lacking data	VA ECMO converted to VV ECMO for lung support	Yes; needed VV ECMO for 3 days	57 days in hospital. Amputation of fingers and metatarsi due to frostbite.	Discharged to rehabilitation facility
McCormac J (2016)[108]	M/39	U; found outdoors a winter night on remote location	No; 21 °C; asystole	CPR for 167 min. VA ECMO for rewarming; VF at 23 °C.	6.97; K+5.7	Weaned off ECMO after conversion of VF to SR	No; extubated the same day	One week in hospital	Full recovery. Discharged to his home
Eckert I (2017) [72]	F/43	U; poorly dressed. exhausted and collapsed when hiking in a snowstorm with her husband	Yes; 20.7 ° C; asystole	CPR for 63 min. VF converted to SR after rewarming to 28 °C.	7.09; K+ 2.6; lactate 10.99	CPB for 2 hrs 5 min to reach 28 °C. Weaned from CPB at 34 °C.	No	2 days in ICU	Fully recovered. No sequela

Carlsen A (2017) [102]	M/14; Swedish citizen	S; canoeing a lake accompanied by two friends; capsized and fell into cold water. Rescued after 63 min	Yes; 18 ° C; asystole	CPR for, at least 82 min. during transportation with ambulance helicopter to UH and connected to CPB.	6.48; K+4.8: lactate 20	VA CPB converted to VA ECMO for lung support	Yes; excessive pulmonary edema. Trans-ported to Karolinska Hospital on ECMO	Weaned off ECMO after 3 days. Extubated after 7 days. Left ICU after 11 days	Discharged to his home 18 months later. Peripheral neuropathy remained
Carlsen A (2017) [102]	M/14; Swedish citizen	S; canoeing a lake near Norwegian boarder with two friends; capsized and fell into cold water. On shore after approximately 125 min	Yes; 14.5 ° C; asystole	CPR with LUCAS2 for during transport with ambulance helicopter to UH in Trondheim where he arrived 185 min after the accident	6.56; K+5.2: lactate 22	VA ECMO from the start. rhabdomyolysis and compartment syndrome both arms and right leg	Yes; continued on ECMO and transported to Karolinska Hospital in Stockholm	Weaned off ECMO after 5 days. Extubated after 12 days. Renal replacement for 18 days. Discharged from ICU 19 days after the accident	Discharged to his home after 2 months; 18 months later, peripheral neuropathy remained with impaired fine motor skills. Walking distance increased
Carlsen A (2017) [73]	M/95; found in the basemen t	U; wet clothes repairing water leak.	Yes; 22.9 ° C; initially bradycardia; VF in ambulance	CPR for 41 min. before start CPB. Converted to SR at 31 °C	6.9; K+ 5.5; lactate 12.5	VA femoral CPB; rewarmed to 36 º C.	No; flail chest and unilateral pneumothorax due to multiple rib fractures after chest compressions	Two days in ICU	Discharged to a nursing home after 13 days and to his home 3 weeks later.
Merz S (2017)[116]	M/1; Found submers ed in a lake, face down	S; cold winters day (March)	No; asystole; 26 °C	CPR for 90 min before ROSC. Transferred to ECMO center after 3 hrs	6.75; K+ 2.9; lactat 17.	ECMO between left common carotid artery and internal jugular vein for 72 hrs	Yes, lung edema	Weaned from ventilator after 16 days and transferred to local hospital. Regressing hemiparesis	Discharged to his home with paresis in regression after 65 days.
Nelson J (2018) [103]	M/1.8	S; discovered by family floating face down in a pond.	Yes; 31.8 ° C	CPR for 14 min. resulted in ROSC, but an escalating need for cardiac support.	6.63; lactate 17.7	Connected to VA ECMO for 5 days. Received dexmede-tomidine for neuroprotection.	Yes; lung edema due to left ventricular failure	Discharged from pediatric ward to rehab after 17 days	Discharged to his home after 26 days with mild sequelae.

Grapatsats K (2018)[104]	M/30	U; drug and alcohol intoxication. Found outdoors at - 5 ° C .	Yes; 25 ° C. CPR started in ambulance	CPR for 45 min until VA femoral ECMO was started	Lacking data	VA ECMO for 6 hrs.	No lung edema. Extubated the next day.	Transferred to the neurology ward after 7 days.	Full recovery.
Lacey A (2018) [105]	M/47	Snowmobile accident	No; 19 ° C	CPR for 210 min before ECMO	Lacking data	VA ECMO. Rewarmed to 30 °C. VF converted to SR	Lacking data	Severe frostbite. Four limb amputations	Discharged to his home without neurological deficits.
Bunya N (2018)[109]	F/29	U; found near snowy mountain trail at air temperature of -2 to +1 °C after intoxication with drugs and alcohol	No; 22 °C; asystole. CPR from site of accident. Bag and mask ventilation because of mandibular rigidity	Successful tracheal intubation with video-laryngoscope upon arrival at hospital. Start mechanical ventilation and VA ECMO	6.82; K+ 5.4;lactate 11.5	Rewarmed with VA ECMO. At 28 °C, VF, which was converted to SR. Weaned off ECMO after 2 days.	No; her condition improved gradually and she was discharged from ICU after 12 days	Transferred to the neurological ward from where she was discharged after 42 days	She recovered with no sequela and confessed that she had intended to commit suicide
Kakizaki R (2018) [111]	F/74	U; found outdoors unconscious in severe hypothermic condition.	Yes; temperature not registered. CA in ambulance and CPR started immediately.	VA ECMO started upon arrival to hospital for rewarming and resuscitation	No laboratory data. BP dropped suddenly on arrival to ICU. Angiography: normal coronary arteries. Left ventricle dis-played Takotsubo pattern.	Weaned from ECMO day 3 and extubated day 5	Excessive catecholamine secretion during hypothermia regarded as the most likely pathogenic mechanism underlying takotsubo cardiomyopathy.	Discharged from ICU day 8.	She was discharged from hospital with no sequela. ECMO provides useful circulatory support in patients with takotsubo.
Scandroglio A (2018)[112]	M/14	S; rescued after drowning in a river with water temperature of 15 °C, in which he had been trapped at 2 m	Yes; 29.5 °C; asystole; manual CPR started. Tracheally intubated immediately	VA ECMO and intra-aortic balloon pumping (IABP) were initiated approximately 100 min after he	7.26; K+ 2.7; lactate > upper detection limit; base excess -20; DIC. Renal failure (create-nine 249	After 4 days, VA ECMO and IABP were disconnected. The fifth day, cerebral magnetic resonance showed thalamic ischemia	No lung edema noted. After 13 days, he was awake and neurologically intact.	Discharged from hospital after 37 days. He went back to school after few months and speaks the four languages he spoke before the accident	A follow-up examination two years after the accident confirmed the absence of neurological deficits although he had an episode of seizures 10 months after the accident

		depth for 43 min.	after rescue. Advanced CPR during transfer by helicopter to nearest UH.	was rescued by firefighters.	µmol/L. Also acute liver failure (ASAT 4925 U/L, total bilirubin 107.6 µmol/L)	and intracranial hypertension. Right inferior leg was amputated due to ischemia. He was transferred to the neurosurgical ICU.			requiring antiepileptic medication.
Fister M (2019) [113]	M/54	I; slipped from a path down into a creek with water temperature of 6 °C	No; VF from the site of accident. Core temperature 25.4 °C. when reaching hospital 2 hrs and 11 min later	VA ECMO started 2 hrs 54 min after hospital arrival. ROSC after defibrillation 1 hrs and 22 min later.	pH 6.78; lactate 10.3; K+ 4.0; left ventricular hypokinesia. Mild troponin increase. Non ST-elevation myocardial infarction.	Weaned from ECMO after 11 hrs and 56 minutes.	Yes; Lung edema. Weaned from mechanical ventilation after 2 days.	Recovered gradually. Discharged from ICU to the ward after 17 days.	Discharged from hospital after 3[illegible] days with normal cardiac functi[illegible] without neurological and cognitive deficits.
Hougardi L (2019) [121]	M/36	U; found unconscious in the forest. Previous history of attempted suicide. Suspected of alcohol and paracetamol overdose. Intubated and mechanically ventilated by arriving health personnel.	No; 23.4 °C; asystole from site of accident. Acetylcystein prophylactically.	CPR for 240 min while attempting to rewarm him non-invasively and while connecting him to VA ECMO. Converted from VF when rewarmed to core temperature of 32 °C.	Acidosis pH (?); K+ (?). He suffered rhabdomyolysis (CPK> 29.000 U/L) but had no acute renal failure. Despite the paracetamol intoxication, he suffered no liver failure.	VA ECMO until day 5.	Yes; aspiration pneumonia.	Stayed in ICU from 23. February to 6.March.	After 17 days in hospital, he was discharged with no neurological deficits.
Forti A (2019)[114]	M/31	U; rock climber caught by thunderstorm at 2,800 m altitude and cooled while climbing a vertical wall. Evacuated in a helicopter winch operation.	Yes; 26 °C; upon landing diagnosed with VF. Tracheally intubated, mechanical ventilation star-ted. CPR with a chest compression device for 3	VA ECMO started 4 hrs and 10 min after he was found unconscious. ROSC was achieved after 8 hrs and 42 min of HCA.	Forti A et al. (2019)	6.97; K+ 4.8; lactate 14.9; Renal replacement therapy from day 1 because of release of inflammatory metabolites, myoglobin, and creatine phosphokinase.	Day 7 VA ECMO was changed to VV ECMO because left ventricle function had improved while respiratory failure still remained.	Yes; respiratory failure. Weaned off VV ECMO day 10.	Mechanical ventilatory support until he was extubated day 21. Only mild retro-grade amnesia at day 28.

			hrs and 42 min from site of accident until connection to ECMO.						
Vicinanza A (2019)[110]	M/6	U; arrived at hospital with severe hypothermia.	Yes; 27.2 °C. Non-invasively rewarmed over 8 hrs. Combined malnutrition and beriberi.	VA ECMO started because of pulse-less electrical activity 840 min after arrival to hospital	Compensated metabolic acidosis; lactate 5.5; CK-MB 2.262 UI/L; myoglobin 1.978 ng/mL; troponin 684 ng/L	VA ECMO from 14 hrs after arrival to 6th day of hospital stay	Yes; lung edema. Weaned from mechanical ventilation after 6 days.	Underwent total parenteral nutrition with vitamin supplementation in pediatric ICU.	Full recovery
Mendonca M (2019) [117]	M/7	S; fell 40 m into a canyon and was immersed for > 20 min in a cold mountain creek from where he was rescued. External rewarming started immediately after rescue.	Yes; 24.4 ° C; found with palpable bradycardic pulses on rescue. Bradypnenic; Tracheally intubated and, mechanically ventilated from site of accident	HCA occurred during ambulance helicopter landing at the trauma center. ECG showed VF and PEA.	pH 7.3; K+ 2.4 mmol/l; lactate 4 mmol/l	VA ECMO started after landing. VF converted to sinus rhythm when body core temperature had reached 29 °C. Then, temperature was kept at 34 °C for 14 hrs;	Yes; respiratory failure due to lung edema. Weaned off VA ECMO after 48 hrs.	Multiple Intracranial hematomas were removed	On discharge from hospital after 35 days, paresis of the right-facial nerve and the right leg remained as only sequelae.
Riera J (2020) [118]	F/34	U; Collapsed during a mountain trek. HCA from site of accident until reaching hospital after 2 hrs and 25 min.	Yes; 18°C; Unresponsive, asystolic, pulse-less, unreactive dilated pupils.	HCA for 2 hrs and 25 min until reaching hospital	pH 6.81; lactate 10.3; K+ 4.4 mmol/L	VA ECMO Rewar-med at a rate of 3 °C/hr. VF when reaching 30 °C. Then, successfully Defibrillated and obtained ROSC after 6 hrs and 10 min.	Yes: lung edema; weaned off ECMO after 45 hrs and 40 min.	Temperature was kept at 34 °C for 14 hrs. Left ICU after 6 days. Discharged from hospital after 11 days.	A month after the episode, she had resumed her normal activities of daily living.

Ledoux A (2020) [119]	M/46	U; found on the street a winter day with coma , but still breathing	Yes; 22.4 °C; cardio-vascular instability-ty, diagnosed with VF during mobilization.	HCA for 45 min. CPR from the scene of accident lasting totally 260 min until VA ECMO was established. ROSC obtained after 7 direct current shocks.	pH 6,98; lactate 14.2 mmol/L; K+ 4.4 mmol/L, Fibrinogen monomers 122 ug/mL; D-Dimers 15.1 mg/L; CPK 6700 U/L	VA ECMO rewarming started after 260 min of attempted circulatory stabilization. A BT of 36 °C was reached after 40 min.	Yes; Pneumonia (Pseudomonas aeruginosa). Left ventricle EF was 55% the second postoperative day. Weaned off ECMO after 2 days.	Generalized myoclonic seizures after stopping sedation day 4. Extubated day 17. Left ICU day 26.	Left hospital after 40 days. After 2 months, he was fully conscious and oriented, had no seizures and walked without help.
Kosinski S (2020) [120]	M/54	U; found lifeless in the forest a winter morning. Medical personnel unaware that the patient had an ICD to prevent sudden cardiac death.	No; initial temperature unknown. Upon arrival to hospital a core BT of 17.8 °C was measured.	HCA from 0915. CPR for 159 min, from 09.18. Diagnosed with not shockable cardiac rhythm and intubated from the place he was found.	pH 7.14; K+ 3.5 mmol/L	After VA ECMO rewarming to a BT of 18.4 °C, a 15 J shock with the ICD restored SR with subsequent stimulation at 50 beats/min	No;	Analysis of the ICD recordings revealed 18 tachyarrhythmic events on the day the patient was found.	The patient survived and was discharged from hospital without neurological sequelae.
Beaton C (2020) [122]	M/46	U; found pulseless in a snowbank at an outdoor temperature of - 5 ° C. He had agonal respiration. CPR started immediately	No; initially, VF on the ECG, which changed to asystole after 1 mg epinephrine and DC shock.	CPR including tracheal intub-ation and mechanical ventilation from scene of accident. Arrived at hospital with ambulance helicopter after 28 min. BT 22 °C. CPR for 150 min.	pH .7; K+ 4.7; capillary glucose 14.7 mmol/L	VA ECMO after 150 min of CPR. After 20 min of ECMO and a core BT of 24.9, the monitor showed conversion to VF. He was successfully defibrillated to sinus bradycardia.	Yes; ICU stay was complicated by aspiration pneumonia, acute kidney failure and rhabdomyolysis. He was weaned off ECMO after 7 hrs and extubated after 5 days.	Discharged .neurologically intact from ICU after 6 days	He was discharged from hospital with good neurological outcome 15 days post admission.
#Mroczek T et al. (2020) [123]	M/2.25	U; left home between 3 and 4 AM. Found lifeless, barefoot and without trousers 620 m from home at outdoor temperature of - 5 ° C at 9 AM.	No; initial temperature unknown. Upon arrival to hospital initial BT was 12.6 °C and dropped to 11.8 °C 10 min. later.	CPR by paramedics started 09.24 AM. Due to bodily stiffness, prehospital intubation was deferred.	pH?; K+ 4.9; lactate 5.2, glucose 19.5 mmol/L.	ST ECMO from 11.08 AM after 103 min of CPR. He resumed ECG activity at BT of 17 ° C and weaned from ECMO after 24 hrs. He was extubated after 36 hrs.	Yes; ICU stay was complicated by pneumonia.	Magnetic resonance imaging of the head performed 24 h after weaning from ECMO did not show any abnormalities	Discharged to rehabilitation after 9 days. , The boy was very active and discharged home after 64 days. Currently, 5 years after the accident, the boy lives normally with small limitations of movements.

*In these patients cardiopulmonary bypass (CPB) was used after a period of external rewarming and successful cardiopulmonary resuscitation (CPR); BT, body temperature; NA, not appropriate; SR, sinus rhythm; ST, sternotomy; VA, veno-arterial; VV, veno-venous; NIRS, near infrared spectroscopy; UH, University Hospital; ST, mid-sternal thoracotomy. #A reviewer made us aware of this case report from November 2020. Our last literature update was 28.08.20.
